# A Comparative Study of the *Arabidopsis thaliana* Guard-Cell Transcriptome and Its Modulation by Sucrose

**DOI:** 10.1371/journal.pone.0049641

**Published:** 2012-11-21

**Authors:** George W. Bates, David M. Rosenthal, Jindong Sun, Maitreyi Chattopadhyay, Emily Peffer, Jing Yang, Donald R. Ort, Alan M. Jones

**Affiliations:** 1 Department of Biological Science, Florida State University, Tallahassee, Florida, United States of America; 2 Global Change and Photosynthesis Research Unit, Agricultural Research Service, United States Department of Agriculture, Institute for Genomic Biology, University of Illinois, Urbana, Illinois, United States of America; 3 Pioneer Hi-Bred International, Johnston, Iowa, United States of America; 4 Department of Cell Biology and Molecular Genetics, University of Maryland, College Park, Maryland, United States of America; 5 Department of Plant Sciences, University of California Davis, Davis, California, United States of America; 6 Departments of Biology and Pharmacology, University of North Carolina, Chapel Hill, North Carolina, United States of America; University College Dublin, Ireland

## Abstract

Microarray analysis was performed on RNA isolated from guard cells that were manually dissected from leaves of *Arabidopsis*. By pooling our data with those of two earlier studies on *Arabidopsis* guard cell protoplasts, we provide a robust view of the guard-cell transcriptome, which is rich in transcripts for transcription factors, signaling proteins, transporters, and carbohydrate-modifying enzymes. To test the hypothesis that photosynthesis-derived sugar signals guard cells to adjust stomatal opening, we determined the profile of genes expressed in guard cells from leaves that had been treated with sucrose. The results revealed that expression of 440 genes changed in guard cells in response to sucrose. Consistent with this hypothesis, these genes encoded cellular functions for photosynthesis and transport of sugars, water, amino acids, and ions. Plants of T-DNA insertion lines for 50 genes highly responsive to sucrose were examined for defects in guard cell function. Twelve genes not previously known to function in guard cells were shown to be important in leaf conductance, water-use efficiency, and/or stomate development. Of these, three are of particular interest, having shown effects in nearly every test of stomatal function without a change in stomatal density: *TPS5* (At4g17770), a TRAF domain-containing protein (At1g65370), and a WD repeat–containing protein (At1g15440).

## Introduction

The guard cell is arguably the most dynamic cell type in higher plants. At the start of the light period, guard cells actively extrude protons, driving K^+^ accumulation and stomatal opening, a process that is reversed at day’s end. During the day, guard cells integrate signals, principally transpiration rate, internal CO_2_ concentration, light, and ABA, and adjust stomatal aperture from moment to moment to balance the plant’s competing need of water retention for turgor against the needs of evaporative cooling and carbon fixation. Although ion transport clearly has a key role in stomatal movements, guard-cell carbohydrate metabolism also has a central role. During the day, starch in the guard-cell chloroplasts is broken down to produce malate to balance cytoplasmic pH and, along with Cl^–^, serve as a counter ion for K^+^ accumulation [1]. Sucrose also accumulates in guard cells during the light period and is a major osmotic contributor to determining stomatal aperture [2]. Although guard cells are capable of photosynthetic carbon reduction, they have insufficient chlorophyll content and photosynthetic capacity to be self supporting and therefore must import sugars to supply the bulk of their carbon and energy needs [3].

Sugars are not only sources of carbon and energy but are also regulators and integrating signals in a wide range of basic plant processes extending from embryogenesis and seedling growth to flowering and senescence [4], [5]. Sugars may also play diverse roles in guard-cell function. The production of sugars has been proposed to be regulated in the leaf by negative feedback from high levels of photosynthate, which inhibit transcription of genes encoding photosynthetic enzymes, thus providing carbon balance between source (e.g., mesophyll) and sink (e.g., epidermal) tissues [6], [7]. By sensing intercellular CO_2_ levels in the leaf and adjusting stomatal aperture, guard cells modulate photosynthetic rates and thus are also involved in the balance between source and sink at the whole-plant level. Guard cells must respond to water availability. In part, this response is achieved through abscisic acid (ABA) signaling, but guard cells also respond to vapor pressure deficit and do so by monitoring transpiration rate [8]. In fact, some evidence supports a model for the regulation of stomatal aperture through sucrose accumulation in the guard-cell apoplast under conditions of high transpiration rate [9], [10]. According to this model, under conditions of high transpiration rate in homobaric leaves, photosynthate is swept from the mesophyll cells to the guard cells’ apoplast by the transpiration stream and is deposited there when water evaporates from the leaf. Thus, the accumulation of photosynthate, specifically sucrose, provides a signal for reduction of stomatal aperture. Changing levels of sucrose in the guard-cell apoplast provide a fine-tuning mechanism to balance the competing needs for CO_2_ uptake for photosynthesis and for control of water loss through evapotranspiration: When the vapor pressure deficit is large and/or excess photosynthate is present in the leaf because of low sink demand, sucrose is deposited at the guard-cell apoplast and results in stomatal closure, reduced rates of photosynthesis, and reduced water loss. The reverse occurs when the pressure deficit is small and/or sucrose levels in the leaf are low because of high sink demand. This model for coupling photosynthetic rates and evapotranspiration applies only to apoplastic phloem loaders [11] with homobaric leaf anatomy. Supporting evidence for the model comes from study of *Vicia faba* guard cells, which load sugars from the aploplast via the phloem as does *Arabidopsis* [12]. When transpiration rates are high, the levels of sucrose in the guard cell apoplast reach 150 mM and this sugar accumulation is correlated with midafternoon decreases in stomatal aperture [13], [14].

**Figure 1:**
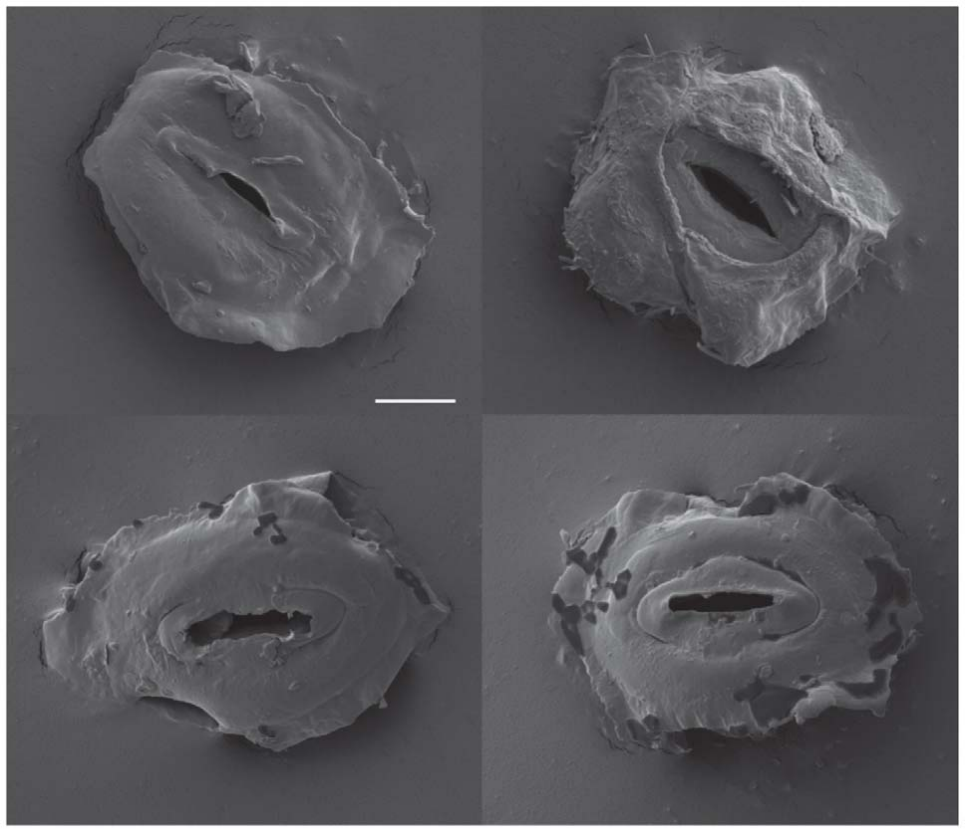
Images of guard cell pairs. Scanning electron microscope images of guard-cell pairs isolated by dissection from freeze-dried leaf tissue. Scale bar, 5 µm.

**Figure 2:**
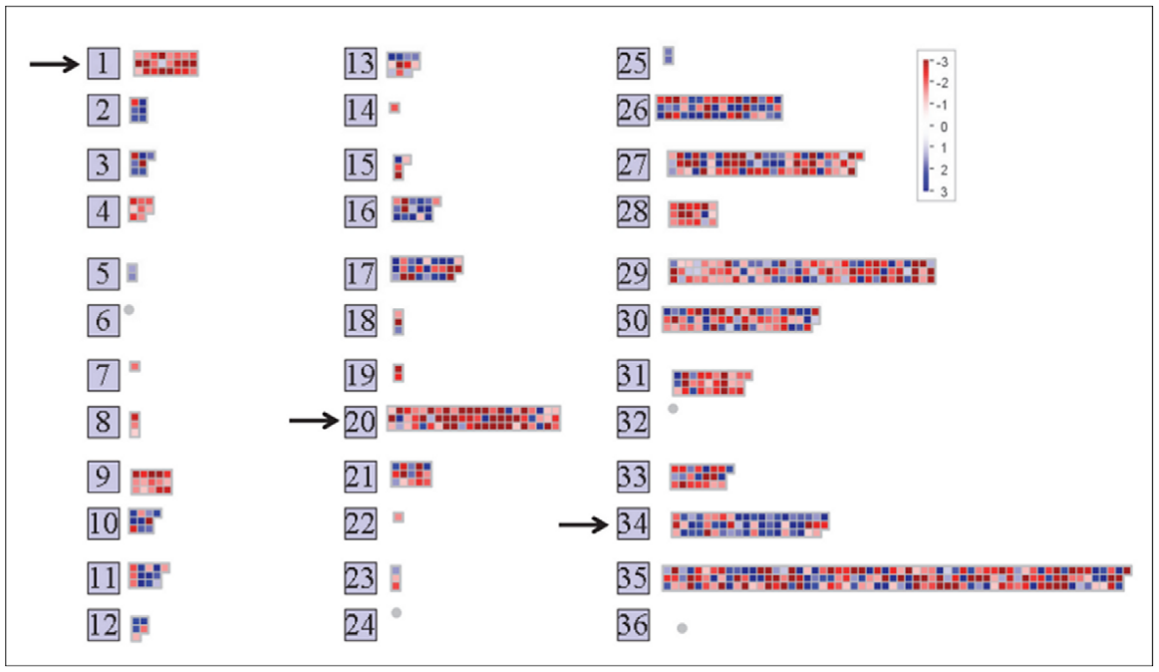
Graphical display from the MapMan program showing differences in gene expression between intact guard cells and guard cell protoplasts. The data displayed here are all those genes listed in Table S3. The view is from MapMan’s “Overview” of gene expression, the genes are grouped into functional categories. Genes up-regulated in intact guard cells are in blue, those up-regulated in guard cell protoplasts are in red. Functional categories that are significantly different (Wilcoxon rank sum test with Benjamini Hochberg correction) between the two guard cell preparations are indicated by the arrows. Group 1 is photosynthesis, group 20 is stress, group 34 is transcription and RNA modifying enzymes. Several other groups of genes that appear different between the two protoplast preparations, but which are not statistically different (probably because of the small number of genes in those groups), are group 10 (cell wall), group 9 (mitochondrial electron transport), group 4 (glycolysis), group 3 (minor carbohydrate metabolism), and group 31 (cell organization).

Although high levels of extracellular sucrose impose an osmotic effect promoting stomatal closure, extracellular sugars may also be signals that modulate expression of genes involved in guard-cell movements. Investigations of sugar-response mutants showed that ABA, ethylene, and sugar-response pathways overlap extensively [15]. Sugars affect genome-wide changes in gene expression [16]–[22]. They activate expression of genes encoding sugar metabolism and energy-storage functions, and they repress sugar-production functions, but effects are also found on genes involved in nitrogen metabolism, stress and defense, and hormone signaling.

**Table 1:** The fifty genes that are most strongly up-regulated in guard cell protoplasts compared with intact guard cells.

AGI Gene Identifier	SI Intact Guard Cells	SI Guard Cell Protoplasts	Fold Difference	Gene Name/Description
At3g46230*	4.4	12.9	364.9	ATHSP17.4 (HEAT SHOCK PROTEIN 17.4)
At5g12030*	4.8	12.7	236.2	AT-HSP17.6A (HEAT SHOCK PROTEIN 17.6A)
At1g59860*	5.5	12.8	164.2	17.6 kDa class I heat shock protein)
At2g29500*	4.2	11.4	147.7	17.6 kDa class I small heat shock protein
At2g46240*	4.7	11.9	144.3	BAG6 (BCL-2-ASSOCIATED ATHANOGENE 6)
At5g12020*	4.5	11.6	132.5	HSP17.6II; 17.6 kDa class II heat shock prot.
At4g12400*	3.9	10.6	102.8	stress-inducible protein, putative
At1g16030*	5.4	12.1	100.9	Hsp70b (heat shock protein 70B)
At4g25200*	4.3	11.0	99.8	ATHSP23.6-MITO (heat shock protein 23.6)
At5g48570	6.0	12.7	97.5	peptidyl-prolyl cis-trans isomerase, putative
At5g51440*	5.9	12.4	90.7	23.5 kDa mitochondrial heat shock protein
At1g53540*	5.6	12.0	82.5	17.6 kDa class I small heat shock protein
At3g28740	4.4	10.6	76.5	CYP81D1; electron carrier/monooxygenase
At5g52640*	6.8	13.0	73.0	ATHSP90.1 (HEAT SHOCK PROTEIN 90.1)
At1g74310*	7.7	13.9	72.3	ATHSP101 (HEAT SHOCK PROTEIN 101)
rpl23	5.7	11.8	66.3	–
At3g12580*	7.8	13.8	62.8	HSP70 (heat shock protein 70)
psbH	5.0	10.8	56.6	–
psaA	5.7	11.3	50.4	–
At3g01830*	6.7	12.3	48.5	calmodulin-related protein, putative
At1g54050*	6.4	12.0	47.9	17.4 kDa class III heat shock protein
At1g22810*	5.7	11.2	44.9	AP2 domain transcription factor, putative
At2g40350*	6.0	11.4	44.2	DNA binding/transcription factor
At2g20560*	6.1	11.4	39.4	DNAJ heat shock family protein
At5g25450	7.0	12.3	38.1	cytochrome C reductase protein, putative
At1g06760	7.2	12.4	35.2	histone H1, putative
At1g20693	5.6	10.7	34.3	HMGB2 (HIGH MOBILITY GROUP B 2)
At3g28210	6.3	11.4	32.3	PMZ; zinc ion binding
At3g16050*	6.9	11.9	31.0	A37; protein heterodimerization
At5g35320	6.6	11.6	31.0	hypothetical protein
At3g24500*	7.8	12.7	30.6	MBF1C/transcription coactivator
At1g55920	8.2	13.1	29.4	ATSERAT2;1 (serine O-acetyltransferase)
At3g55500*	7.6	12.4	28.8	ATEXPA16 (EXPANSIN A16)
At5g12340	5.3	10.1	27.5	hypothetical protein
At5g47830	5.3	10.0	26.2	hypothetical protein
psbD	6.4	11.0	23.4	–
At2g32190	6.9	11.5	23.2	hypothetical protein
At4g34135	8.5	13.0	22.9	UGT73B2; UDP-glucosyltransferase
At2g02230	6.6	11.1	22.7	AtPP2-B1 (Phloem protein 2-B1)
At3g10020	7.3	11.8	22.6	hypothetical protein
psbE	7.6	12.1	22.4	–
At2g15480	7.6	12.1	22.0	UGT73B5 (UDP-glucosyl transferase 73B5)
At2g01180	7.1	11.5	21.5	ATPAP1; phosphatidate phosphatase
At2g19310	6.2	10.6	21.5	hypothetical protein
At1g30070	6.8	11.2	21.3	SGS domain-containing protein
At3g09350	8.3	12.7	21.3	beta-catenin repeat family protein
At2g37430	7.0	11.4	21.0	zinc finger (C2H2 type) family protein
At5g09590*	7.8	12.1	20.8	MTHSC70-2 (MITOCHONDRIAL HSP70 2)
ycf10_cemA	6.2	10.5	20.3	–
At3g23990*	6.5	10.8	19.3	HSP60 (HEAT SHOCK PROTEIN 60)

Signal intensities (SI) are normalized log2 values. Asterisks identify stress-responsive genes.

The goal of our study was to examine global changes in gene expression in *Arabidopsis* guard cells in response to sucrose and to identify candidate genes for further study. The three previous reports that addressed the guard-cell transcriptome used guard-cell protoplasts as the source of guard-cell RNA [23]–[25]. The present study differs from those by dissecting guard cells from leaves thereby avoiding the high osmoticum and prolonged digestion in cellulytic enzymes that are needed for protoplast isolation and the associated changes in gene expression that accompany those treatments. Specifically, RNA was isolated from guard cells manually dissected from freeze-dried leaf strips. We show that this approach yields a profile of guard cell-gene expression that has some important differences from the profile obtained from guard cell protoplasts. By pooling our results with those of two earlier studies on guard cell protoplasts, we obtained a robust view of the guard cell transcriptome. We identified over 1200 transcripts that are preferentially expressed in guard cells, including a wide range of transporters, transcription factors, and genes for proteins involved in signaling and posttranslational protein modification. The functional roles of many of these genes in guard-cell biology have yet to be evaluated. In our study of guard cells treated with sucrose, we identified over 400 sucrose-responsive genes, again including many transcription factors and genes involved in signaling, but also genes for carbohydrate metabolism, some of which may play regulatory roles in guard-cell movements. To test our hypothesis that some of these sucrose-responsive genes are critical for normal guard-cell function, we evaluated 50 T-DNA-insertion mutations in genes identified in the discovery phase. Guard-cell functional defects were found in 12 mutants, of which none was in a gene previously known to have a role in guard cell biology.

**Table 2:** Selected examples of genes preferentially expressed in guard cells.

AGI Gene Identifier	Guard Cell SI	Leaf SI	Fold Difference	Gene Name/Description
**Regulation of Transcription**				
At2g38300	10.2	4.0	74.8	DNA binding/transcription factor
At1g08810	10.3	5.4	30.9	MYB60 (myb domain protein 60)/transcription factor
At5g05410	12.3	7.4	29.5	DREB2A; DNA binding/transcription factor
At4g05100	10.3	5.9	22.0	AtMYB74 (myb domain protein 74); transcription factor
At3g28910	10.7	6.4	19.0	MYB30 (MYB DOMAIN PROTEIN 30); transcription factor
At2g40260	9.0	4.8	18.6	myb family transcription factor
At3g26744	11.4	7.5	15.0	ICE1 (INDUCER OF CBF EXPRESSION 1); transcription factor
At1g01250	8.3	4.5	13.8	AP2 domain-containing transcription factor, putative
At5g67180	7.8	4.1	13.8	AP2 domain-containing transcription factor, putative
At1g77450	10.8	7.1	12.9	anac032 (NAC domain protein 32); transcription factor
At1g62300	11.1	7.5	12.8	WRKY6; transcription factor
At4g21440	9.1	5.4	12.8	ATMYB102 (MYB-LIKE 102); transcription factor
At3g24140	10.4	6.8	12.6	FMA (FAMA); transcription factor
At1g12610	8.6	5.5	8.3	DDF1; transcription factor
At5g63790	12.0	9.2	7.1	ANAC102 (NAC domain protein 102); transcription factor
At1g12860	9.3	6.5	7.0	SCRM2 (SCREAM 2); DNA binding/transcription factor
At2g46830	9.1	6.4	6.5	CCA1 (circadian clock associated 1); transcription factor
At4g01250	10.7	8.2	5.8	WRKY22; transcription factor
**Signaling and hormone perception**				
At1g62400	11.7	4.9	111.7	HT1 (high leaf temperature 1); protein kinase
At1g11340	10.5	4.8	54.7	S-locus lectin protein kinase family protein
At1g11410	11.5	5.8	49.7	S-locus protein kinase, putative
At2g46070	11.7	6.1	47.1	MPK12 (MITOGEN-ACTIVATED PROTEIN KINASE 12)
At4g24480	10.2	5.2	32.9	serine/threonine protein kinase, putative
At2g30500	11.2	6.2	31.8	kinase interacting family protein
At3g22840	10.2	5.2	31.8	ELIP1 (early light-inducible protein); chlorophyll binding
At5g53890	10.4	5.5	29.4	leucine-rich repeat transmembrane protein kinase
At4g14480	9.8	5.0	26.1	protein kinase family protein
At2g21880	10.0	5.4	22.8	ATRAB7A; GTP binding
At2g40180	10.4	6.0	20.9	ATHPP2C5; protein serine/threonine phosphatase
At1g26600	8.8	4.5	19.0	CLE9 (CLAVATA3/ESR-RELATED 9); receptor binding
At5g20270	11.3	7.1	18.1	HHP1 (HEPTAHELICAL TRANSMEMBRANE PROTEIN1)
At1g07570	11.3	7.2	17.8	APK1A; kinase/protein serine/threonine kinase
At4g33950	11.4	7.3	17.4	OST1 (OPEN STOMATA 1); serine/threonine kinase
At5g07280	9.2	5.1	16.9	EMS1 (EXCESS MICROSPOROCYTES1); protein kinase
At3g24720	9.2	5.2	16.1	protein kinase, putative
At3g49260	10.1	6.3	14.2	iqd21 (IQ-domain 21); calmodulin binding
At2g40120	8.7	4.9	13.8	protein kinase family protein
At3g17790	10.1	6.5	12.4	PAP17; protein serine/threonine phosphatase
At2g24540	9.8	6.1	12.4	AFR (ATTENUATED FAR-RED RESPONSE)
At5g43020	8.5	4.9	12.1	leucine-rich repeat transmembrane protein kinase
At5g06750	10.7	7.1	11.9	protein phosphatase 2C family protein
At5g14640	10.1	6.6	11.1	SK13 (SHAGGY-LIKE KINASE 13); protein kinase
At5g57050	9.6	6.2	10.5	ABI2 (ABA INSENSITIVE 2); serine/threonine phosphatase
At3g14720	10.4	7.0	10.4	ATMPK19; MAP kinase
At4g30610	8.6	5.2	10.3	BRS1 (BRI1 SUPPRESSOR 1); serine-type carboxypeptidase
At3g18040	10.3	7.0	9.7	MPK9 (MAP KINASE 9); MAP kinase
At5g24270	9.2	6.0	9.4	SOS3/calcium-dependent serine/threonine phosphatase
At1g80080	7.5	4.2	9.4	TMM (TOO MANY MOUTHS); protein binding/receptor
At1g74740	8.9	5.8	8.7	CPK30; calmodulin-dependent protein kinase
At4g37580	8.2	5.1	8.3	HLS1 (HOOKLESS 1); N-acetyltransferase
At1g31930	10.5	7.6	7.2	XLG3 (extra-large GTP-binding protein 3)
At2g40940	10.4	7.9	5.7	ERS1 (ETHYLENE RESPONSE SENSOR 1); protein kinase
At5g15230	11.6	9.2	5.1	GASA4 (GAST1 PROTEIN HOMOLOG 4)
At4g26080	11.3	9.0	4.7	ABI1 (ABA INSENSITIVE 1); serine/threonine phosphatase
At4g37590	9.2	7.0	4.5	NPY5 (NAKED PINS IN YUC MUTANTS 5); signal transducer
At1g01560	10.5	8.4	4.5	ATMPK11; MAP kinase/kinase
**Transport**				
At3g25620	11.3	5.4	56.8	ABC transporter family protein
At3g53720	12.4	6.7	54.8	ATCHX20 (CATION/H+ EXCHANGER 20)
At1g12480	10.6	4.9	53.4	OZS1 (OZONE-SENSITIVE 1); transporter
At4g18050	9.9	4.2	51.6	PGP9 (P-GLYCOPROTEIN 9); ATPase, transporter
At1g24400	10.7	5.4	38.4	LHT2 (LYSINE HISTIDINE TRANSPORTER 2)
At3g47750	9.0	3.8	37.6	ABCA4 (ATP binding cassette family A4)/transporter
At3g52310	10.7	5.5	37.1	ABC transporter family protein
At4g18290	9.9	4.8	34.4	KAT2 (POTASSIUM CHANNEL IN ARABIDOPSIS THALIANA 2)
At2g29940	9.4	4.3	33.1	PDR3 (PLEIOTROPIC DRUG RESISTANCE 3); transporter
At5g46240	10.4	5.6	26.4	KAT1 (POTASSIUM CHANNEL IN ARABIDOPSIS THALIANA 1)
At2g47000	9.8	5.1	26.2	ABCB4 (ATP BINDING CASSETTE subfamily 4 transporter)
At5g48485	12.1	7.5	25.1	DIR1; lipid transporter
At5g37500	9.3	4.7	24.2	GORK (gated outwardly rectifying K+channel)
At2g24520	10.3	6.0	19.6	AHA5 (Arabidopsis H(+)-ATPase 5); ATPase
At2g28260	9.2	4.9	19.6	ATCNGC15; calmodulin binding/cation channel
At1g28010	9.0	5.1	15.5	PGP14 (P-GLYCOPROTEIN 14); ATPase, transporter
At3g05030	9.7	6.1	12.3	NHX2 (SODIUM HYDROGEN EXCHANGER 2)
At1g17840	11.2	7.9	9.4	WBC11; ATPase/fatty acid transporter
At2g38940	8.3	5.1	9.0	ATPT2; phosphate transporter/sugar:hydrogen symporter
At4g18910	10.5	7.4	8.8	NIP1;2; transmembrane transporter/water channel
At5g44110	9.1	6.0	8.6	POP1; CER6
At3g02850	7.4	4.5	7.4	SKOR; cyclic nucleotide binding/potassium channel
At1g51500	11.2	8.5	6.6	CER5 (ECERIFERUM 5); ATPase, ABC transporter
At3g23430	7.8	5.4	5.3	PHO1 (phosphate 1)
At3g19930	11.9	9.8	4.5	STP4 (SUGAR TRANSPORTER 4)
**Carbohydrate metabolism**				
At4g12430	10.2	5.4	29.3	TPPF, trehalose-6-phosphate phosphatase
At4g24040	12.1	7.5	24.6	TRE1 (TREHALASE 1); alpha,alpha-trehalase/trehalase
At4g02280	8.7	4.5	18.6	SUS3 (sucrose synthase 3); UDP-glycosyltransferase
At2g21590	7.9	4.0	15.2	APL4; glucose-1-phosphate adenylyltransferase
At2g22240	10.5	6.7	14.5	MIPS2; myo-inositol-3-phosphate synthase
At1g78580	8.3	5.5	7.0	ATTPS1 (trehalose 6-phosphate sysynthase )
At4g22590	11.4	8.6	6.9	TPPG trehalose-6-phosphate phosphatase
**Enzymes**				
At3g55710	9.0	4.5	22.3	UDP-glucoronosyl/UDP-glucosyl transferase family prot.
At4g37870	12.7	9.0	12.4	PCK1 (PHOSPHOENOLPYRUVATE CARBOXYKINASE 1)
At2g26250	12.4	8.9	10.8	KCS10 (3-KETOACYL-COA SYNTHASE 10); acyltransferase
At5g43330	10.1	6.7	10.8	malate dehydrogenase, cytosolic, putative
At4g37370	10.4	7.2	9.2	CYP81D8; electron carrier/monooxygenase/
**Cell wall and cuticle**				
At2g47240	10.2	5.5	26.2	long-chain-fatty-acid–CoA ligase family protein
At4g24510	9.8	6.2	12.9	CER2 (ECERIFERUM 2); acyl transferase
At4g18280	11.9	8.4	11.5	glycine-rich cell wall protein-related
At1g63180	9.5	6.0	11.5	UGE3 (UDP-D-glucose/UDP-D-galactose 4-epimerase 3)
At1g26770	9.4	6.2	9.1	ATEXPA10 (ARABIDOPSIS THALIANA EXPANSIN A 10)
At5g57800	11.2	8.4	7.4	CER3 (ECERIFERUM 3); binding/oxidoreductase
**Other**				
At5g66400	12.3	6.1	74.5	RAB18 (RESPONSIVE TO ABA 18)
At1g64950	10.4	5.4	33.4	CYP89A5; electron carrier/monooxygenase
At1g44760	10.1	5.7	20.9	universal stress protein (USP) family protein
At5g06760	8.7	4.7	16.3	LEA group 1 domain-containing protein
At2g32120	9.9	6.0	15.0	HSP70T-2 (HEAT-SHOCK PROTEIN 70T-2); ATP binding
At1g52080	8.6	4.8	14.2	AR791; actin binding
At5g07990	9.0	5.2	14.2	TT7 (TRANSPARENT TESTA 7); flavonoid 3-monooxygenase

The average normalized log2 signal intensity (SI) for all genes on the arrays (calls present or marginal) was 8.8.

**Table 3:** The 50 most abundant guard cell transcripts.

AGI Gene Identifier	Guard Cell SI	Leaf SI	Fold Difference	Gene Name/Description
At5g15960	13.63	12.53	2.13	KIN1
At1g22690	13.16	8.82	20.28	gibberellin-responsive protein, putative
At4g02890	12.96	11.54	2.68	UBQ14; protein binding
At4g05050	12.91	11.61	2.46	UBQ11 (UBIQUITIN 11); protein binding
At2g05540	12.89	9.01	14.69	glycine-rich protein
At4g37870	12.67	9.03	12.43	PCK1 (PHOSPHOENOLPYRUVATE CARBOXYKINASE 1)
At1g11260	12.64	11.51	2.20	STP1 (SUGAR TRANSPORTER 1)
At2g17840	12.56	10.77	3.45	ERD7 (EARLY-RESPONSIVE TO DEHYDRATION 7)
At2g43520	12.56	7.52	32.89	ATTI2; serine-type endopeptidase inhibitor
At1g67090	12.53	13.66	−2.18	RBCS1A (ribulose bisphosphate carboxylase 1A)
At2g31570	12.48	10.83	3.15	ATGPX2 (GLUTATHIONE PEROXIDASE 2)
At4g18950	12.45	8.97	11.17	ankyrin protein kinase, putative
At3g53720	12.43	6.66	54.75	ATCHX20; sodium:hydrogen antiporter
At2g18960	12.42	11.21	2.32	AHA1 (ARABIDOPSIS H+ ATPASE 1); hydrogen-exporting ATPase
At2g26250	12.37	8.93	10.84	KCS10 (3-KETOACYL-COA SYNTHASE 10); acyltransferase
At3g43720	12.33	9.10	9.40	protease inhibitor/seed storage/lipid transfer protein
At2g46720	12.32	6.72	48.54	KCS13 (3-KETOACYL-COA SYNTHASE 13); acyltransferase
At1g33811	12.31	6.71	48.75	GDSL-motif lipase/hydrolase family protein
At5g05410	12.31	7.43	29.49	DREB2A; transcription factor
At1g79040	12.30	13.77	−2.76	PSBR (photosystem II subunit R)
At1g56580	12.30	8.23	16.80	hypothetical protein
At5g66400	12.28	6.06	74.54	RAB18 (RESPONSIVE TO ABA 18)
At5g62350	12.27	11.08	2.28	invertase/pectin methylesterase inhibitor family protein
At2g34430	12.26	13.51	−2.37	LHB1B1; chlorophyll binding
At3g61470	12.26	13.69	−2.69	LHCA2; chlorophyll binding
At2g05070	12.25	13.45	−2.31	LHCB2.2; chlorophyll binding
At4g23630	12.23	10.98	2.38	BTI1 (VIRB2-INTERACTING PROTEIN 1)
At4g38420	12.21	7.02	36.60	sks9 (SKU5 Similar 9); copper ion binding/oxidoreductase
At2g45820	12.17	10.80	2.59	DNA-binding protein, putative
At2g38310	12.16	10.05	4.31	hypothetical protein
psaB	12.15	13.48	−2.52	–
At5g48485	12.11	7.46	25.10	DIR1; lipid binding/lipid transporter
At5g52310	12.09	10.12	3.92	LTI78 (LOW-TEMPERATURE-INDUCED 78)
At4g24040	12.08	7.46	24.57	TRE1 (TREHALASE 1); alpha,alpha-trehalase/trehalase
At5g63790	12.03	9.20	7.06	ANAC102 (NAC DOMAIN PROTEIN 102); transcription factor
At5g61820	11.99	9.86	4.39	hypothetical protein
At4g32940	11.95	9.98	3.91	GAMMA-VPE; cysteine-type endopeptidase
At3g19930	11.94	9.77	4.52	STP4 (SUGAR TRANSPORTER 4); monosaccharide transporter
At5g54770	11.94	12.98	−2.07	THI1; protein homodimerization
At4g18280	11.92	8.39	11.55	glycine-rich cell wall protein-related
At2g37540	11.91	9.37	5.81	short-chain dehydrogenase/reductase (SDR) family protein
At3g57020	11.91	7.62	19.57	strictosidine synthase family protein
atpB	11.89	13.66	−3.42	–
At1g29660	11.87	8.65	9.31	GDSL-motif lipase/hydrolase family protein
At4g10340	11.87	13.51	−3.13	LHCB5 (LIGHT HARVESTING COMPLEX OF PHOTOSYSTEM II 5)
At2g42600	11.83	10.16	3.18	ATPPC2 (PHOSPHOENOLPYRUVATE CARBOXYLASE 2)
At4g00360	11.83	9.06	6.84	CYP86A2 (CYTOCHROME P450 86 A2); fatty acid hydroxylase
At3g62420	11.83	10.56	2.41	ATBZIP53; transcription factor
At5g25840	11.82	6.46	40.86	hypothetical protein
At3g54890	11.82	13.57	−3.37	LHCA1; chlorophyll binding

Signal intensities (SI) are normalized log2 values. Positive fold differences indicate genes that are preferentially expressed in guard cells compared with the leaf.

## Methods

### Plant Material and Sugar Treatments for Microarray and Real-time PCR Experiments

Plants of *Arabidopsis thaliana* ecotype Col-0 were grown at 22°C, 60% relative humidity (RH), under fluorescent lights (150 µE m^–2^s^–1^, 8 h light/16 h dark diurnal cycles) on Superfine Germinating Mix (FaFard). Mature leaves were harvested from plants 8–10 weeks old that had not undergone the transition to flowering. Plants were grown in batches every 2 to 3 weeks. Each sample of guard cells was taken from an individual plant. Two biological replicates were obtained from plants of different batches that were grown in the incubator at overlapping times. The third biological replicate was from a plant in a batch grown 3 to 6 months later than the first two replicates. With a scalpel, the midrib was removed, and the leaf blade was cut into strips 1 mm wide. The leaf strips were floated on 12.5 mL of a solution of 50 mM KCl +0.1 mM CaCl_2_+10 mM MES-NaOH (pH 6.1) +/−150 mM sucrose or mannitol in a 125-mL Erlenmeyer flask. Interstitial air was removed under vacuum (25 KPa for 30 sec), and the leaf strips were incubated (with swirling) for 5 h at 22°C, 30 µE m^–2^s^–1^ fluorescent illumination. After incubation, the leaf strips were blotted dry, frozen in liquid nitrogen, freeze-dried for 4 days under a vacuum of <10 mm of mercury, and stored under vacuum at –20°C. Guard-cell pairs and mesophyll tissue were manually dissected from the leaf strips at 20°C, 50% RH, under a dissecting microscope at 50× magnification, with scalpels made from fragments of single-edge razor blades. The dissections were done in the laboratory of W. H. Outlaw Jr., who developed this method [26], [27]. Hereafter we will call guard cells isolated by this process “intact guard cells” to distinguish them from guard cells isolated by protoplasting.

**Table 4:** Selected genes in guard cells that responded to sucrose. Signal intensities are normalized log2 values.

AGI Gene Identifier	Signal Intensity in Mannitol	Fold Change due to Sucrose	Gene Name/Description
**Photosynthesis**			
At3g50820	7.2	−17.5	PSBO-2/PSBO2 (PHOTOSYSTEM II SUBUNIT O-2); oxygen evolving
At1g51400	9.3	−6.3	photosystem II 5 kD photosystem II 5 kd protein
At5g64040	9.8	−4.0	PSAN/(photosystem I reaction center subunit PSI-N); calmodulin binding
At2g28000	8.6	3.8	RuBisCO subunit binding-protein alpha subunit, chloroplast
At3g56650	4.9	−3.7	thylakoid lumenal 20 kDa protein
At3g16140	11.0	−2.4	PSAH-1/(photosystem I subunit H-1)
At2g30570	13.4	−2.0	photosystem II reaction center W (PsbW) protein-related
**Transporters**			
At1g61800	5.5	146.2	GPT2/glucose-6-phosphate/phosphate translocator, putative
At4g01010	9.0	−15.7	CNGC13/cyclic nucleotide-regulated ion channel, putative
At2g48020	10.3	−6.5	sugar transporter, putative
At1g61570	8.0	6.2	TIM13/mitochondrial import inner membrane translocase
At4g36670	13.3	−4.4	mannitol transporter, putative
At1g71880	12.2	−3.4	SUC1/sucrose transporter/sucrose-proton symporter
At3g19930	14.1	−2.2	STP4/sugar transport protein
At4g00430	13.0	2.0	TMP-C/plasma membrane intrinsic protein, putative
**Transcription factors and RNA regulation**			
At5g49450	11.5	−29.4	BZIP1/bZIP family transcription factor
At3g44750	4.7	25.5	HD2A/histone deacetylase, putative
At1g66390	5.1	12.9	PAP2/myb family transcription factor, putative
At1g56110	6.7	10.8	NOP56 (ARABIDOPSIS HOMOLOG OF NUCLEOLAR PROTEIN NOP56)
At5g53290	3.7	6.8	AP2 domain-containing transcription factor, putative
At4g14540	8.2	−5.3	NF-YB/CCAAT-box binding transcription factor subunit B
At1g14920	5.9	5.2	GAI (GA INSENSITIVE); transcription factor
At1g03110	3.6	5.2	transducin family protein/WD-40 repeat family protein
At3g16770	14.8	−4.7	RAP2.3/AP2 domain-containing protein RAP2.3
At1g08460	9.6	−3.3	HDA8/histone deacetylase family protein
At1g43160	13.6	−3.3	RAP2.6/AP2 domain-containing protein
**Signaling and posttranslation modifications**			
At5g21170	10.0	−24.9	5′-AMP-activated protein kinase beta-2 subunit, putative
At2g44130	9.4	−22.5	kelch repeat-containing F-box family protein
At3g59940	11.3	−21.5	APG4b/autophagy 4b
At1g18350	6.6	10.0	mitogen-activated protein kinase kinase (MAPKK), putative (MKK7)
At1g48630	8.5	8.8	RACK1B/guanine nucleotide-binding family protein
At3g18130	6.5	8.3	RACK1C/guanine nucleotide-binding family protein
At4g38470	10.1	−7.9	protein kinase family protein
At3g10530	4.8	7.2	transducin family protein/WD-40 repeat family protein
At2g38760	6.5	6.2	ANN3/annexin 3/calcium binding, phospholipid binding
At5g39030	9.1	−5.4	protein kinase family protein
At1g80440	13.5	−5.4	kelch repeat-containing F-box family protein
At1g50920	7.5	3.7	GTP-binding protein-related
At2g01570	7.0	2.0	RGA1/gibberellin response modulator
**Carbohydrate metabolism and glycolysis**			
At5g56870	12.4	−380.9	beta-galactosidase, putative/lactase, putative
At4g17770	6.4	50.3	TPS5/trehalose-phosphatase family protein
At3g62410	10.8	−30.5	CP12 domain-containing protein
At4g09020	6.9	19.1	ISA3/isoamylase, putative/starch debranching enzyme, putative
At2g18700	12.0	−9.6	TPS11/trehalose-phosphatase family protein
At1g62660	9.8	−9.1	BFRUCT3/beta-fructosidase/invertase, vacuolar
At4g39210	8.5	6.5	APL3/ADP-glucose pyrophosphorylase large subunit
At5g20250	13.5	−6.4	DIN10/raffinose synthase family protein
At4g09510	6.6	4.7	CINV2/invertase neutral, putative
At3g03250	9.4	4.4	UGP1/UDP-glucose pyrophosphorylase, putative/UGPase, putative
At1g55120	7.7	4.3	ATFRUCT5/(BETA-FRUCTOFURANOSIDASE 5)
At2g36390	8.1	4.2	SBE2-1; starch branching enzyme class II
At3g06500	12.7	−3.1	beta-fructofuranosidase, putative/neutral invertase, putative
At5g03650	8.6	2.9	SBE2.2/(STARCH BRANCHING ENZYME 2.2)
At5g20280	9.4	2.2	sucrose-phosphate synthase 1F
At4g29220	10.6	−2.0	PFK1/phosphofructokinase family protein
**Cell Wall**			
At5g49360	12.8	−410.0	BXL1/glycosyl hydrolase family 3 protein/xylosidase
At3g10740	9.6	−8.5	ASD1/arabinofuranosidase
At4g32410	9.5	2.2	CESA1/cellulose synthase, catalytic subunit, putative
At1g12780	11.2	−3.1	UGE1/UDP-glucose 4-epimerase/UDP-galactose 4-epimerase
**Redox**			
At5g49730	9.9	−30.1	ferric reductase-like transmembrane component family protein
At3g22460	9.6	−12.2	cysteine synthase, putative/O-acetylserine sulfhydrylase, putative
At1g03850	10.5	−11.7	glutaredoxin family protein
At1g11530	10.4	−7.5	thioredoxin family protein
**Enzymes**			
At5g24160	8.9	−30.8	SQP1,2/squalene monooxygenase 1,2/squalene epoxidase 1,2
At1g73600	6.9	20.8	NMT3/phosphoethanolamine N-methyltransferase 3, putative
At1g03090	9.7	−17.1	MCCA/methylcrotonyl-CoA carboxylase alpha chain, mitochondrial
At1g62540	7.0	17.0	flavin-containing monooxygenase family protein/FMO family protein
At1g55020	9.9	−12.7	LOX1/lipoxygenase1
At5g08570	6.9	8.8	pyruvate kinase, putative
At4g34200	10.1	6.2	D-3-phosphoglycerate dehydrogenase, putative/3-PGDH, putative
**Other**			
At4g35770	10.5	−141.8	SEN1/DIN1/senescence-associated protein
At5g22920	10.7	−55.5	zinc finger (C3HC4-type RING finger) family protein
At1g05340	9.4	−37.4	expressed protein
At1g80130	5.1	36.0	expressed protein
At4g18630	4.7	24.5	expressed protein
At4g27450	11.2	−21.1	expressed protein
At1g52930	5.2	15.0	brix domain-containing protein
At5g23850	5.5	11.9	expressed protein
At5g58650	8.3	−9.7	expressed protein
At1g65370	3.8	8.8	meprin and TRAF homology domain-containing protein
At4g34950	9.0	8.4	nodulin family protein
At1g01770	9.3	−8.1	expressed protein
At4g13750	5.2	6.9	NOV/No Vein/auxin response

FDR <0.05 for all genes shown. Positive fold changes for genes up-regulated in sucrose compared with mannitol, negative fold changes indicate down regulation in sucrose compared with mannitol. Signal intensity is the normalized log2 signal intensity for each gene in the mannitol treatment (calls present in at least one treatment, sucrose or mannitol). The average normalized log 2 signal intensity on the arrays (calls present) was 7.2.

### RNA Isolation

RNA was isolated from dissected guard cells as well as from mesophyll tissue dissected from freeze-dried leaves, with the PicoPure RNA Isolation Kit (Arcturus). Samples of approximately 10 to 15 µg of mesophyll tissue, or of 50–100 guard cells, were used for RNA isolation. The samples were collected in a small plastic boat, 10 µL of extraction buffer was added, and after a quick pipetting up and down, the sample was loaded into a 0.2-mL centrifuge tube, and RNA was purified according to the manufacturer’s instructions, including treatment with DNase during the isolation.

**Figure 3:**
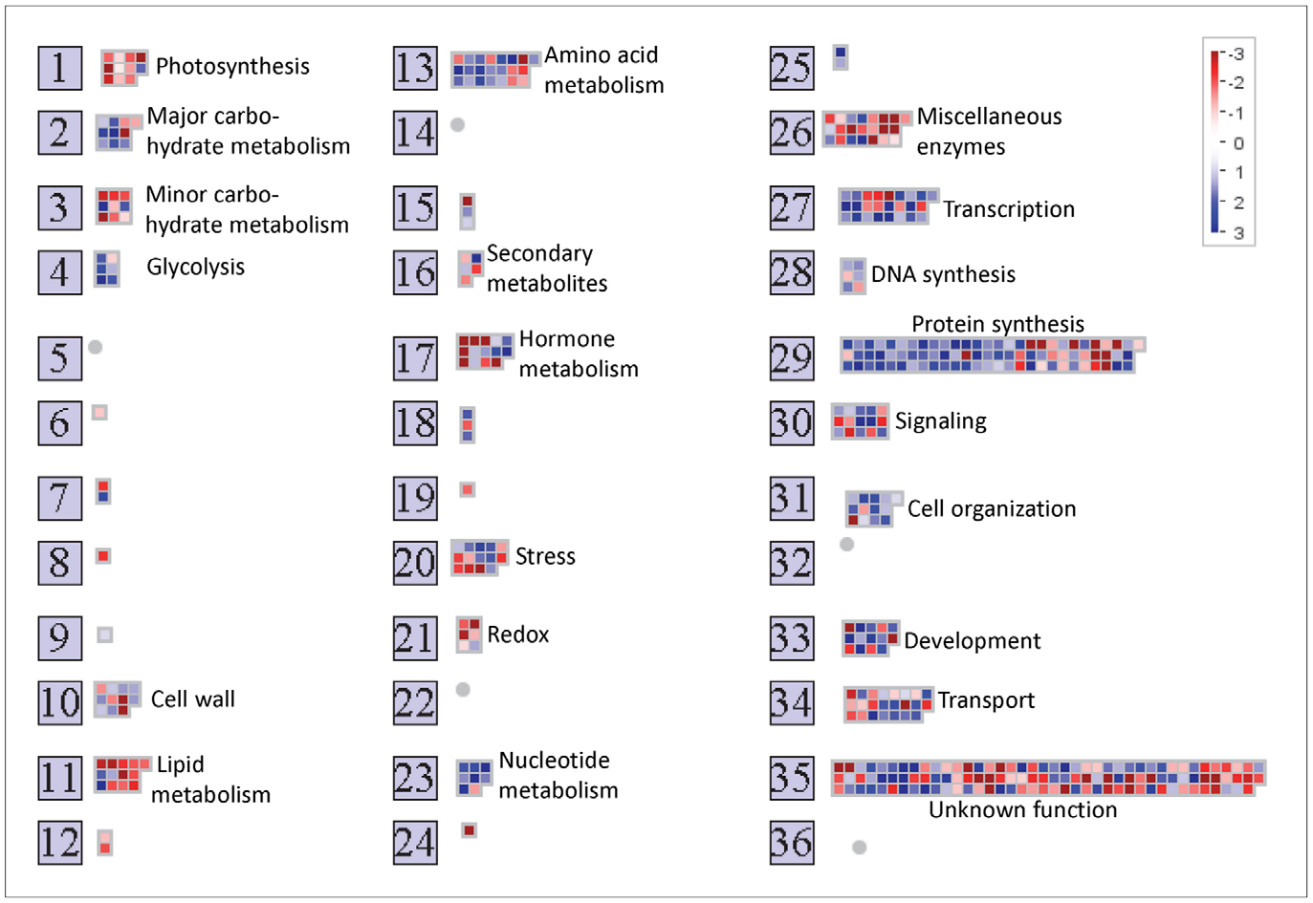
Graphical display from the MapMan program showing sucrose-responsive gene expression in guard cells. The data displayed are those listed in Table S7. The view shown is from MapMan’s “Overview” of gene expression, the genes are grouped into functional categories. Genes up-regulated by sucrose (versus mannitol) are in blue, those down-regulated by sucrose (versus mannitol) are in red.

### Reverse Transcription and Real-time PCR

Five hundred ng of RNA was used for reverse transcription. First-strand cDNA synthesis was performed with Invitrogen’s Superscript II (Life Technologies) according to the manufacturer’s instructions. For unamplified RNA, cDNA synthesis was primed with oligodT; cDNA synthesis from amplified RNA used gene-specific primers. After reverse transcription, *E. coli* RNase H (2 U µL^−1^) was added to digest the RNA from RNA:cDNA hybrids.

Real-time PCR reactions were conducted in a 25-µL reaction volume with the QuantiTect SYBR Green PCR Kit (Qiagen) and 2 µL of the 20-µL cDNA reaction. Real-time PCR was carried out on an ABI 7500 Fast Real Time PCR System (Life Technologies) with the following thermocycler program: 15 min at 95°C and 40 cycles of 15 s at 94°C, 45 s at 57°C, 60 s at 72°C. Production of a single product was verified by melting-curve analysis. For the experiment in which the effects of RNA amplification on different genes was examined, the real-time PCR data were expressed on an absolute basis by reference to standard curves for each gene. The standard curves were produced by PCR performed on a dilution series of purified PCR product for each gene. In real-time PCR used to confirm gene-expression changes on the microarrays, the data for each gene were normalized to expression of *CCH1* (*CONDITIONAL CHLORINA*, At5g13630) as a control (ΔΔCt method). *CCH1* was chosen because the microarrays showed it was highly expressed in guard cells and its expression was not changed by the sugar treatments used here (sucrose versus mannitol). Two genes commonly used for normalization, actin (*ACT2*, At3g18780) and ribulose bisphosphate carboxylase (*RBCS*, At1g67090), could not be used because their expression was changed by the sugar treatments. PCR efficiency for each gene was determined from the slope of the real-time curve during its exponential phase as described by Ramakers et al. [28]. Primers used in RT PCR are listed in Table S1.

**Figure 4:**
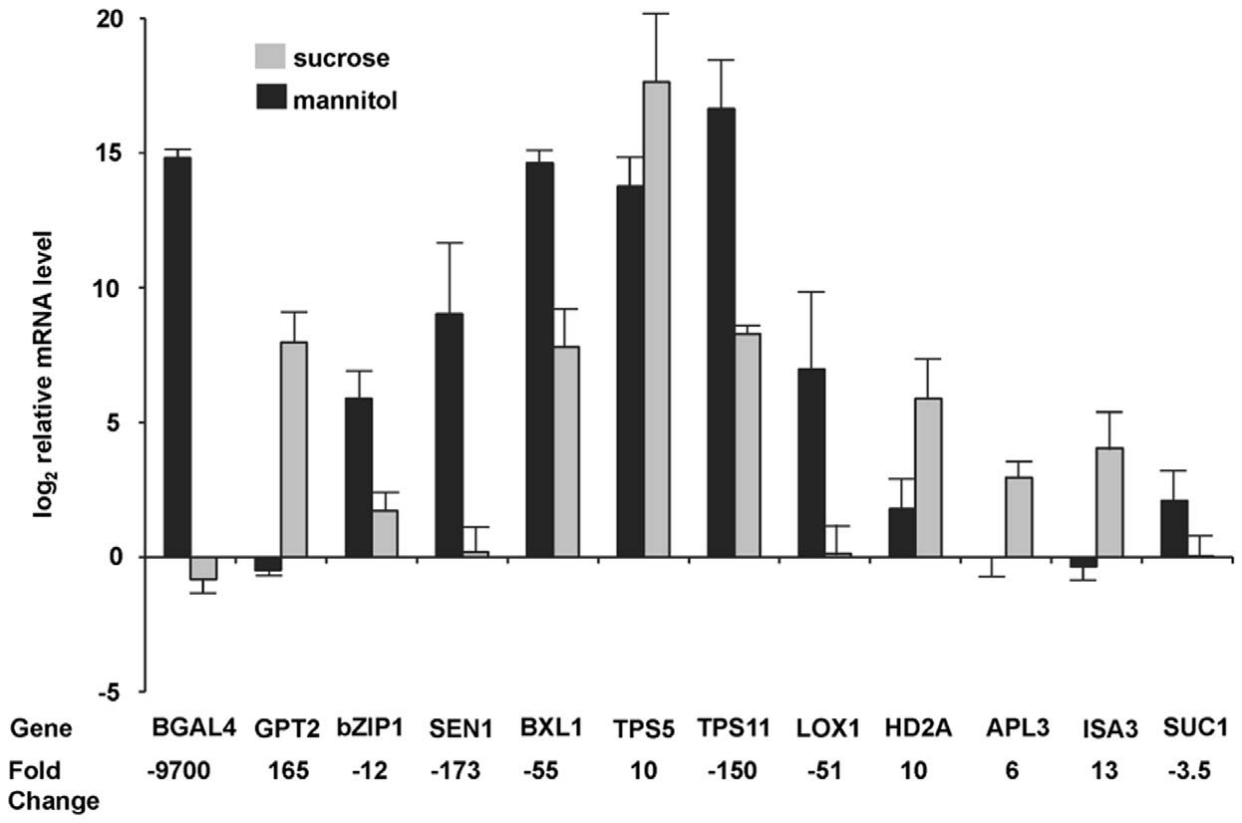
Real-time PCR data for selected genes whose expression responded to sucrose. The data are averaged values for four biological replicate samples (± se) for RNAs of guard cells isolated from leaves treated with either sucrose or mannitol. The data are normalized to the expression of the *CCH1* gene (At5g13630), which is expressed at high levels in guard cells and does not respond to sucrose. Fold changes were calculated from the difference in the log2 relative mRNA level (determined by real-time PCR, not by microarray) for sucrose versus mannitol treated samples. Positive fold changes indicate genes that were up-regulated in sucrose.

### RNA Amplification

RNA amplification was performed with the MessageAmp II aRNA™ Amplification Kit (Ambion) according to the manufacturer’s instructions. Two rounds of RNA amplification were performed on the samples. When RNA from guard cells was amplified, the entire sample (RNA from 50–100 guard cells in 20 µL of elution buffer) was reduced under vacuum to 8 µl and used for amplification. In samples being amplified for microarray analysis, the amplified RNA was labeled by replacement of UTP with biotin-11-UTP according to the manufacturer’s instructions. Typically, 30 µg of amplified RNA was recovered from each guard-cell sample.

### Microarray Slide Hybridization and Scanning

Biotin-labeled, amplified RNA was fragmented, and hybridized to ATH1 GeneChips (Affymetrix) at the University of North Carolina’s Functional Genomics Core Facility according to Affymetrix’s instructions (see Affymetrix user manuals P/N 702232 and P/N 702731). The arrays were washed and stained with R-phycoerythrin streptavidin in the GeneChip Fluidics Station 400. Arrays were scanned with a Hewlett Packard GeneArray Scanner. Affymetrix GeneChip Microarray Suite 5.0 software was used for washing and scanning control and for image analyses. The data are MIAME complaint and have been deposited in the Gene Expression Omnibus (GEO) database (http://www.ncbi.nlm.nih.gov/geo), accession number GSE37408.

### Analysis of Microarrays to Determine the Effects of Sucrose on Guard Cell Gene Expression

The samples used in this comparison were designated GEO samples: GSM918078, GSM918079, GSM918080 (guard cells from wild-type leaves treated with sucrose) GSM918087, GSM918088, GSM918089 (guard cells from *rgs1* leaves treated with sucrose) and GSM918072, GSM918073, GSM918074 (guard cells from wild-type leaves treated with mannitol), GSM918081, GSM918082, GSM918083 (guard cells from *rgs1* leaves treated with mannitol). Statistical tests were performed by the staff at the University of Florida’s Interdisciplinary Center of Biotechnology Research (www.biotech.ufl.edu) with the BioConductor statistical software (http://www.bioconductor.org/), which is an open-source and open-development software for analyzing microarray and other high-throughput data based primarily on the R programming language [29]. The Affymetrix raw data files (CEL files) were imported into the R environment and analyzed by BioConductor packages. Quality assessment for evaluation of overall data coherence was performed with the Affy and AffyQC Report packages, and the raw data were then background-corrected, normalized, and summarized by the GeneChip robust multiarray averaging algorithm [30]. Probe sets that were absent calls in all the arrays were removed from further analysis after normalization. To identify differentially expressed genes, we employed a linear modeling approach and the empirical Bayesian estimate method implemented in the Limma package, which yields a moderated t-statistic for each gene [31]. The p-values were adjusted using the Benjamini and Hochberg method [32] to control the false discovery rate (FDR). A cut-off of FDR <0.05 was used for gene discovery. Annotations were obtained from the Affymetrix web site (http://www.affymetrix.com/estore/index.jsp).

### Analysis of Microarrays for Comparison of the Guard-cell and Leaf Transcriptome

Data from Pandey et al. [25] were obtained from the GEO database accession number GSE19520. Samples GSM486892, GSM486893, and GSM486894 are for guard-cell protoplasts from leaves of untreated wild-type Col-0 plants; samples GSM486916, GSM486917, and GSM 486918 are of leaves of untreated wild-type Col-0 plants. Data from Yang et al. [24] were obtained from ArrayExpress (http://www.ebi.ac.uk/arrayexpress/files) accession number E-MEXP-1443. The samples used were Js33 (guard-cell protoplasts isolated in the presence of transcriptional inhibitors), 1756.Schroeder (guard-cell protoplasts isolated without transcriptional inhibitors), Js35 (mesophyll protoplasts isolated in the presence of transcriptional inhibitors), and 1758.Schroeder (mesophyll protoplasts isolated without transcriptional inhibitors). Data from our own intact guard cells used in this analysis were GEO samples GSM918075, GSM918076, GSM918077, GSM918084, and GSM918085 (guard cells from Col-0 and *rgs1* plants whose leaves were not treated with sugars). Data for mature rosette leaves (Col-0) were obtained from ATGenExpress (http://www.arabidopsis.org/portals/expression/microarray/ATGenExpress.jsp), Expression Atlas of *Arabidopsis* Development; the samples used were ATGE14A, ATGE14B, and ATGE14C. Note, the data of Leonhardt et al. [23] were not included in our comparison because these data were obtained using the early version of the Affymetrix *Arabidopsis* gene chip that had only 8,100 gene probes rather than the ATH1 array (22,400 probes) that were used in the other 3 studies.

**Table 5:** The 12 T-DNA insertion lines that have defects in stomatal movements.

AGI Gene Identifier	SALK T-DNA Insertion Identifier	Fold Change in Response to Sucrose	Gene Name/Description
At4g17770	SALK 144791	50.3	TPS5
At1g03110	SALK 025857	5.2	TRM82 (RNA Modification 82)/WD-40 repeat family protein
At1g15440	SALK 037412	8.5	PWP2/WD-40 protein/CUL4-RING ubiquitin ligase complex
At1g55020	SALK 012188	−12.7	LOX1, lipoxygenase
At1g62540	SALK 098896	17	flavin-containing monooxygenase family protein
At1g65370	SALK 104078	8.8	meprin and TRAF homology domain-containing protein
At3g10530	SALK 057632	7.2	WD-40 protein/CUL4-RING ubiquitin ligase complex
At3g22450	SALK 081267	−12.2	structural component of the ribosome
At4g38470	SALK 112195	−7.9	STY46; serine/threonine/tyrosine kinase 46
At5g39030	SALK 007613	−5.4	protein kinase family protein
At5g49450	SALK 059343	−29.4	bZIP1/transcription factor
At5g49730	SALK 099597	−30.1	FRO6/ferric reductase-like transmembrane protein

The fold changes in response to sucrose are from the microarray data. Positive fold changes indicate up-regulation in sucrose (versus mannitol), negative fold changes indicate down-regulation by sucrose (versus mannitol).

For analysis, CEL files were uploaded into GeneSifter (Geospiza, http://www.geospiza.com) and normalized by robust multiarray averaging. The threshold for identifying genes that were differentially expressed was a 2-fold difference in expression with *t*-test p-value <0.05 and FDR (Benjamini Hochberg) <0.05. The Affymetrix quality calls for each gene were recovered by MAS5 normalization, and any genes that were not “present” (average call <0.75) in the guard cell samples were eliminated.

### Measurement of Photosynthetic Rates and Stomatal Conductance

#### Growth conditions before measurements of gas exchange

Seeds were surface sterilized and cold stratified at 4°C in sterile water for 48 hours and then planted on sterilized potting soil (Sunshine LC1 Mix, Sun Gro Products) in 3- × 3-inch pots. After germination, seedlings were thinned to one plant per pot. Growth-chamber lights were set to deliver a photosynthetic photon flux density of 135±5.8 (sd) µmol m^–2^s^–1^ of photosynthetically active radiation for 10 h. Day and night temperature was 19.4±0.2°C (sd) and chamber RH was kept constant at 76±1.8% (sd). After 14 days of growth, fertilizer (Scotts Miracle Gro Water Soluble All Purpose Plant Food @ 0.5 g per liter) was applied weekly throughout the experiment.

#### Gas exchange methods

Photosynthesis (A) and stomatal conductance (g_s_) were measured with an open gas-exchange system (LI-6400, LI-COR). Plants were taken from growth chambers, and a leaf was immediately placed in the cuvette of the gas exchange system and allowed to reach steady-state photosynthesis at its growth [CO_2_] (390 ppm) at saturating light levels of 700 µmol m^–2^s^–1^. Initial leaf-chamber conditions were set to a constant block temperature (Tblock) of 24°C, the leaf vapor-pressure deficit (VpdL) was less than 1 kPa, and chamber RH was 65%. The plant remained under constant conditions for at least 10 min before steady-state photosynthesis, conductance, and water use efficiency (A/g_s_) were recorded. All measurements were made on 4 to 5 different plants for each genotype.

**Figure 5:**
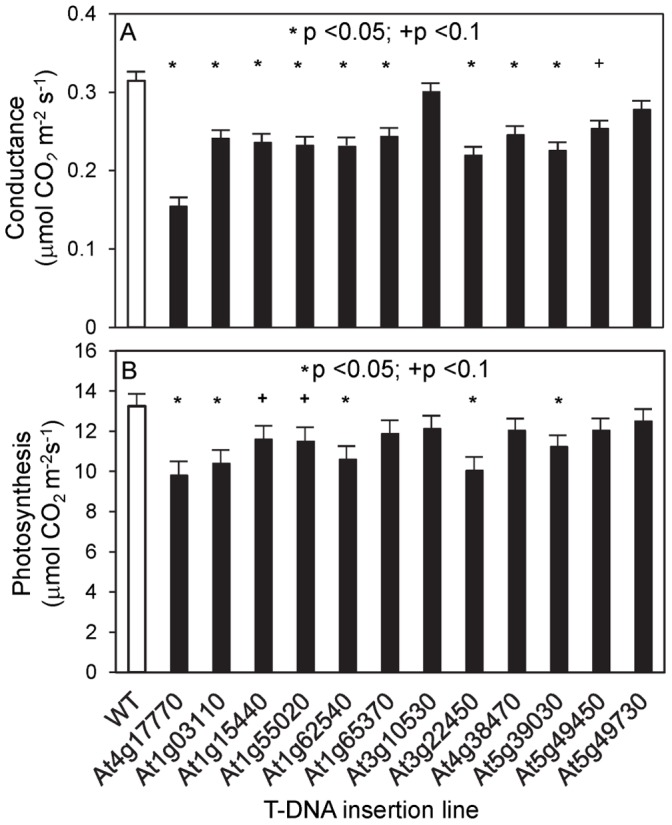
Stomatal conductance of selected T-DNA insertion mutants. Steady-state leaf stomatal conductance (A) and photosynthesis (B) measured under ambient conditions at 24°C, 390 ppm CO_2_, relative humidity 65–70%. The data are averaged values (± se) for 4 to 5 plants for each genotype. The T-DNA insertion lines tested were SALK 144791 or *tps5-1* (At4g17770), SALK 025857 (At1g03110), SALK 037412 (At1g15440), SALK 012188 (At1g55020), SALK 098896 (At1g62540), SALK 104078 (At1g65370), SALK 057632 (At3g10530), SALK 081267 (At3g22450), SALK 112195 (At4g38470), SALK 007613 (At5g39030), SALK 059343 (At5g49450), and SALK 099597 (At5g49730). WT, wild type; _*_ p<0.05;+p<0.1.

The response of stomatal conductance and photosynthesis to two environmental perturbations was measured sequentially, immediately after the steady-state measurement. First, the cuvette [CO_2_] was increased from 390 ppm to 800 ppm within 2 min; immediately after the new [CO_2_] was reached, photosynthesis and stomatal conductance were recorded every 15 s for 10 min. Next, while the chamber [CO_2_] was held at 800 ppm, the RH was quickly lowered by replacement of the air inside the cuvette with dry air. The RH decreased from 65% to an average of less than 21% (VpdL >2) within 2.5 min. After the low RH target was reached, photosynthesis and stomatal conductance were recorded every 15 s for 10 min. The rates of change in A, g_s_, and A/g_s_ over time (i.e., slope of change in g_s_ over 2.5 min) were calculated from at least 10 points once the cuvette conditions had reached the target [CO_2_] or RH values.

### Measurement of Stomatal Density

Impressions were made of the abaxial side of fully-expanded leaves using Aquasil Ultra LV dental impression material (Dentsply International, Milford DE). Clear nail polish was painted on to the leaf impressions, peeled off when dry, and observed at 200X magnification using an Olympus BX61 light microscope. All stomata in the microscope’s camera field of view were counted. Measurements were made on 2 to 4 plants for each genotype and 2 leaves for each plant. Ten separate measurements were made at different positions on each leaf.

## Results and Discussion

### Comparison of Gene Expression Profiles for Guard-cell Protoplasts and Guard Cells Isolated by Dissection

Three previous studies used microarrays to examine the *Arabidopsis* guard cell transcriptome, and in each of these studies the RNA was isolated from guard cell protoplasts [23]–[25]. A concern at the onset of our study was whether gene expression was affected by the extended cell digestion in cellulytic enzymes and high osmotic strength media that is required for protoplast isolation. Therefore, in the present study, guard cells were individually dissected from freeze dried leaves. RNA was isolated from samples of 50–100 guard cell pairs and amplified twice using T7 RNA polymerase to obtain sufficient RNA for microarray analyses. Figure 1 shows examples of guard-cell pairs isolated by dissection. Preliminary studies using RNA isolated from freeze-dried and fresh leaves, and RT-PCR to measure transcript levels, indicated that the conditions needed for freeze-drying and dissection did not result in transcript degradation and that two rounds of amplification with T7 RNA polymerase gave on average 8 × 10^5^–fold amplification with approximately a 2-fold variation among the genes and 30% variation between replicate samples; rare transcripts (*KAT1, POTASSIUM CHANNEL IN ARABIDOPSIS THALIANA 1*) amplified as well as abundant ones (*RBCS, RIBULOSE BISPHOSPAHTE CARBOXYLASE*) (Table S2). Additional evidence that the amplified RNA was of good quality was provided by histograms of the signal intensity on each array (Figure S1A), degradation plots for control probes on the Affymetrix microarrays (Figure S1B), and pairwise comparisons (scatter plots) of all the biological replicate microarrays (Figure S2).

**Figure 6:**
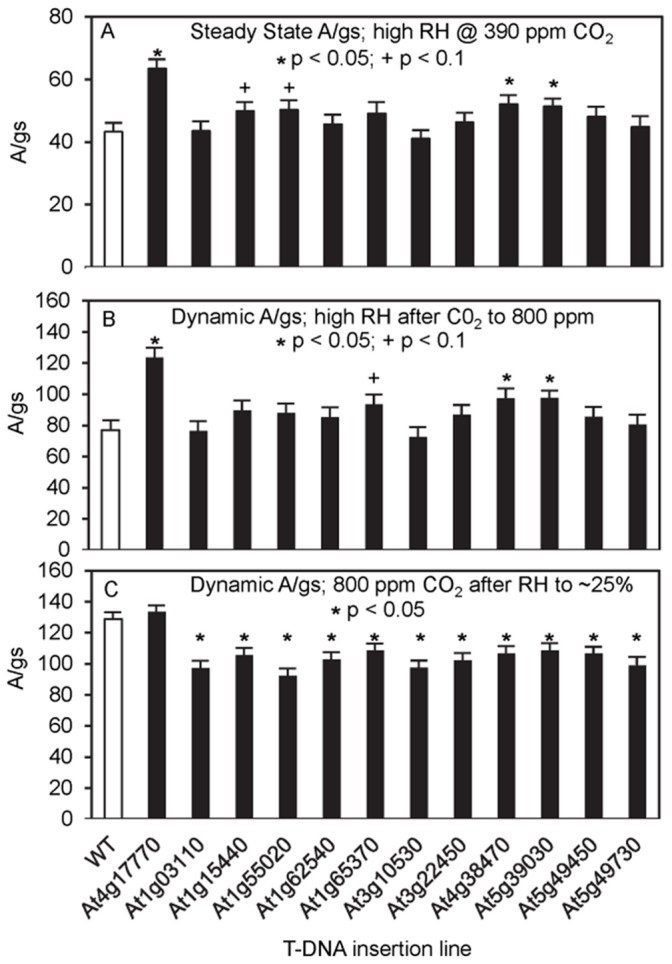
Water-use efficiency of T-DNA insertion mutants. Instantaneous water-use efficiency, the ratio of photosynthesis to conductance (A/g_s_) at 65–70% relative humidity and 390 ppm CO_2_ (A) after a rapid increase of [CO_2_] to 800 ppm (B) and after a rapid decrease in relative humidity to 25% (C). The data are averaged values (± se) for 4 to 5 plants for each genotype. The T-DNA insertion lines tested are as in Figure 5. WT, wild type; _*_ p<0.05;+p<0.1.

Comparison of data from published microarray studies of guard-cell protoplasts [24], [25] with data from an equal number of our arrays for intact guard cells revealed strong statistical evidence for differential expression of 807 transcripts; 518 were up-regulated in the protoplast samples compared with the intact guard cells, and 289 were up-regulated in the intact guard cells compared with the guard cell protoplasts (Table S3). MapMan software [33] was used to categorize gene function. The major differences were in genes annotated for stress, photosynthesis, and transport (Figure 2). Fifty genes from the protoplast samples not found in the intact guard cell samples were related to abiotic stress. These included 37 heat shock-protein genes and genes that respond to cold and or salt stress. Of the 25 genes most strongly up-regulated in the protoplast samples but not in the isolated guard cells, 19 were for stress-related genes as expected given the stressful conditions of protoplasting (Table 1 and Table S3). Twenty-four genes involved in photosynthesis were up-regulated in the protoplast samples but not in the intact guard cells. Most of these genes encode elements of the light reactions, but some encode carbon reduction cycle enzymes, including the large subunit of ribulose bisphosphate carboxylase (*RBCL*), which was 14-fold higher than in protoplasts than intact guard cells. The protoplast samples showed lower expression of transcripts for transporters than did the intact guard cells, possibly because of the high osmotic strength media used in protoplast isolation. These included the disaccharide transporter *SUC1* (down-regulated 8-fold in the guard cell protoplasts compared with the intact guard cells); the aquaporins *PIP1A*, *PIP1B*, and *PIP1D* (down-regulated 8- to 12-fold); and *KAT1* (down-regulated 7-fold), which is known to be preferentially expressed in guard cells [23], [34].

**Figure 7:**
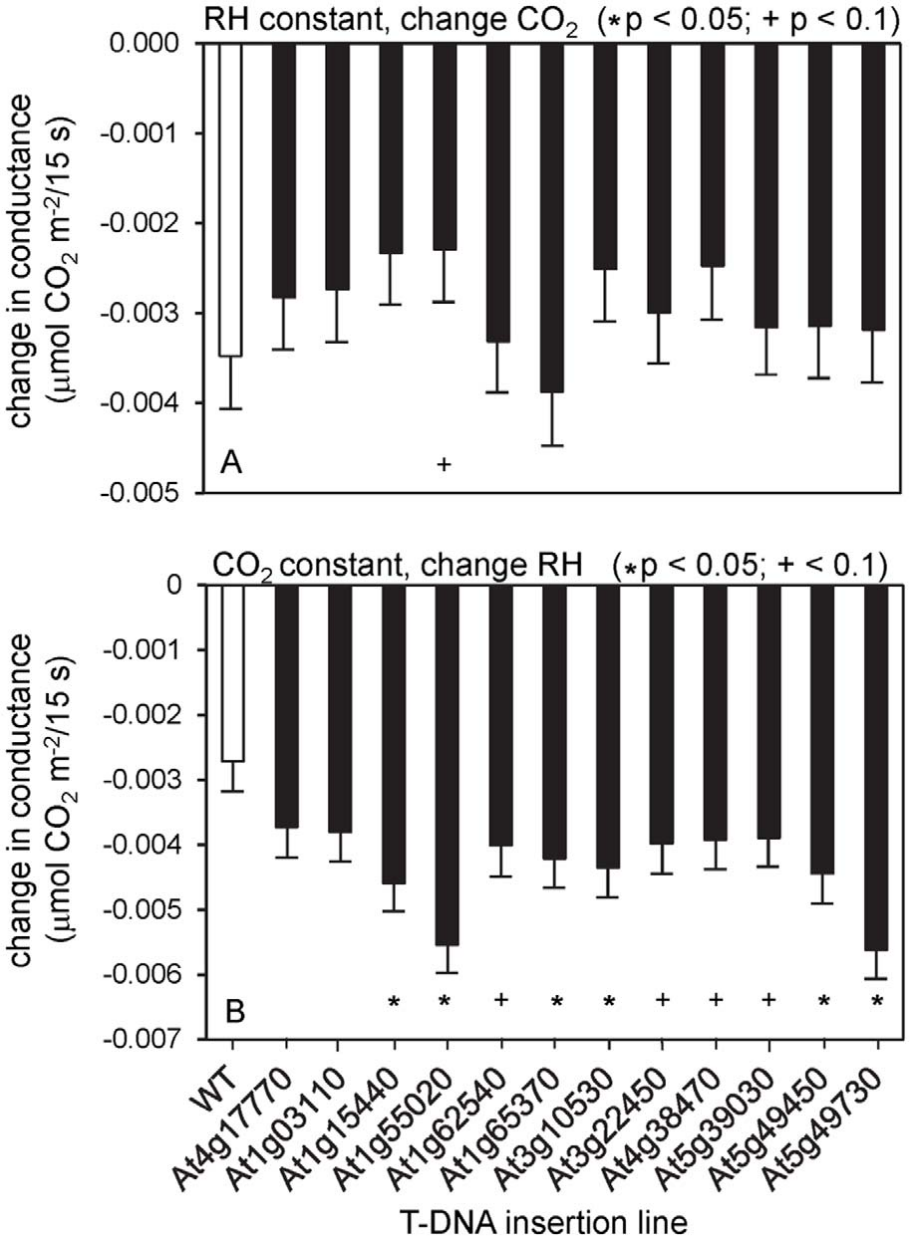
Change in conductance in T-DNA insertion mutants after perturbation of steady-state conditions. (A) Change of [CO_2_] from 400 to 800 ppm (relative humidity held constant at ∼65%). (B) Change of relative humidity to 25% (CO_2_ held constant at 800 ppm). The data are averaged values (± se) for 4 to 5 plants for each genotype. The T-DNA insertion lines tested are as in Figure 5. WT, wild type; _*_p<0.05;+p<0.1.

Although the transcript profiles obtained from guard-cell protoplasts included a set of stress-related genes not found in the intact guard cells, there were a great many more genes that were shared by both protoplast and intact guard cells. For example, of 1337 genes for which expression in guard-cell protoplasts was at least 4-fold higher than that in whole leaves, 70% were also found to show higher expression in intact guard cells vs. whole leaves, and for 99% of these genes, the direction of preferential expression was the same for both types of samples (the genes were up-regulated either in guard cells or in the leaf). The correspondence between the data sets rises to 82% if stress-responsive genes are eliminated (those are almost entirely from the protoplast-derived samples). Microarrays for both intact guard cells and protoplasts also identified genes already known to be up-regulated in guard cells (vs. whole leaf), including *KAT1*, *GORK (*GATED OUTWARDLY-RECTIFYING K^+^ CHANNEL), *HT1 (HIGH TEMPERATURE 1)*, *CHX20 (CATION/H^+^ EXCHANGER 1)*, *SLAC1 (SLOW ANION CHANNEL-ASSOCIATED 1)*, and *ICE1 (INDUCER OF CBF EXPRESSION 1)* [34]–[39].

Four lines of evidence therefore support the reliability of our approach to analyzing guard-cell transcripts. First, preliminary experiments showed that transcripts remained stable during dissection; freeze-dried leaf samples left on the clean-room bench for 0 to 3 days showed no change in actin or *RBCS* transcript levels. Second, two rounds of RNA amplification with T7 RNA polymerase introduced only 2-fold variation among the six genes tested (Table S2), and transcripts present at low levels (*KAT1*) amplified to the same degree as much more abundant transcripts (*RBCS*). One round of RNA amplification is routinely used for preparation of microarray targets, and other studies used two rounds of RNA amplification (for example, Nawy et al. [40]). The degradation plots for Affymetrix control probes on all our microarrays were similar, indicating that the samples were comparable, but the slopes on these plots were steep, as is expected when RNA is amplified twice. Third, many genes shown to be preferentially expressed in guard-cell protoplasts were also preferentially expressed in our guard-cell samples; these include *KAT1* [34], *HT1* [36], *STP1* (*SUGAR TRANSPORTER 1*) [41], *At1g22690* [24], the cytochrome P450 family gene *CYP86A2*, the ABC transporter *PDR3* [42], the MYB family transcription factor *MYB60* [43], *CHX20* [37], the MAP kinases *MPK9* and *MPK12* [44], the SNF1-related protein kinase *OST1* [45], *ICE1* [39], *GORK* [35], and *SLAC1* [38]. Fourth, the microarrays performed with protoplasts and with intact guard cells identified similar sets of genes as being preferentially expressed in guard cells.

### Genes Preferentially Expressed in Guard Cells or in Leaves

Comparison of the pooled data for guard cells (protoplast derived plus those isolated by dissection) with those for rosette leaves revealed that the steady-state levels of 1253 transcripts were at least 2-fold higher in guard cells (FDR <0.05) and that the steady-state levels of 1365 were higher in the leaf. Table S4 provides the full list of genes that were differentially expressed in guard cells versus the leaf, Table S5 lists all the transcripts detected in guard cells, Table 2 gives selected examples of genes preferentially expressed in guard cells, Table 3 lists the 50 most abundant guard-cell transcripts, and Figure S3 shows MapMan’s graphical display of the functional categories of genes differentially expressed in guard cells and the leaf.

Transcripts of genes for all aspects of photosynthesis (light reactions, carbon reduction cycle, photorespiration, chloroplast structure), redox regulators and tetrapyrrole synthesis were 2- to 50-fold higher in the leaf than in guard cells. Guard cells had significantly more transcripts for genes coding for transcriptional regulators (and RNA modifying enzymes), transporters, and mitochondrial electron transport proteins. Guard cells contain fewer chloroplasts than mesophyll cells [3] and depend primarily on the uptake of sugars, rather than photosynthesis, to power metabolism [3], [13]. The greater abundance of transcripts for mitochondrial electron transport proteins supports the view that mitochondria are an important site of ATP production in guard cells [3]. A striking difference between the guard-cell and leaf transcriptomes is the large number of transcripts for genes involved in the regulation of transcription that are at higher steady-state levels in guard cells compared with the leaf. MapMan indentified 221 transcripts for transcription factors or chromatin- or RNA-modifying proteins that are differentially expressed in guard cells versus leaves, and 177 of these are more highly expressed in guard cells than in leaves. Transcription factors make up 14% of the genes preferentially expressed in guard cells, but transcription factors account for only 7% of genes in the *Arabidopsis* genome as a whole [46]. The abundance of transcription factors in guard cells suggests that transcription has a particularly important role in guard-cell responses [see also 47]. Some of these transcription factors have known roles in guard cell differentiation (*ICE1* [39], *FAMA* [48]) and stomatal movements (*MYB60* [43]), but the functions of most are unknown. Guard cells are also rich in transcripts for genes encoding proteins involved in signaling, posttranslational modifications, and ubiquitin-dependent protein degradation. These included MAP Kinases (11), receptor-like kinases (25), and G proteins (15) as well as receptors and ligands potentially involved in development. Among these genes were *HT1*, *OST1*, *ABI1(ABSCISIC ACID INSENSITIVE 1)*, *ABI2*, *MPK9*, and *MPK12*, all which have already been shown to be involved in regulation of stomatal movements [36], [44], [45], [49], and *TMM (TOO MANY MOUTHS)*, which plays a role in guard-cell development [50]. A wide range of signaling pathways were represented, including ABA, calcium, light, CO_2_, and phosphoinositides. Some evidence also indicated that GA and brassinosteroid signaling may have roles in guard-cell movements (*GA INSENSITIVE DWARF 1C*, *GAST1 PROTEIN HOMOLOG 4*, *BRI1 SUPRESSOR 1*, *SQUALENE EPOXIDASE 3*, and *STEROL METHYLTRANSFERASE 3* transcripts are all up-regulated).

Ion transport is critical for stomatal movements and guard cells use a set of ion transporters distinct from those in the leaf. Not surprisingly, transcripts for the potassium channels, *KAT1 KAT2*, *GORK* and *SKOR*, were abundant and preferentially expressed in guard cells as were the cation/H^+^ transporter *CHX20* and the anion transporter *SLAC1* (*OZS1*). These transporters are known to be important in stomatal opening and closure [37], [38], [51], [52]. Other ion transporters more strongly expressed in guard cells than the leaf included *AHA5* (proton-ATPase), *NHX2* (Na^+^/H^+^ exchanger), *CNGC15* (cyclic nucleotide gated potassium channel). Twenty nine genes coding for ABC-transporter-family proteins were much more strongly expressed in guard cells than in leaf (see Table S6 for a list), and a number of these were among the most abundant guard-cell transcripts. Some of these ABC transporters are known to be involved in formation of the cuticle (*CER5*, *WBC11*), a feature of guard cells but not mesophyll cells, while the functions of the others are unknown.

**Figure 8:**
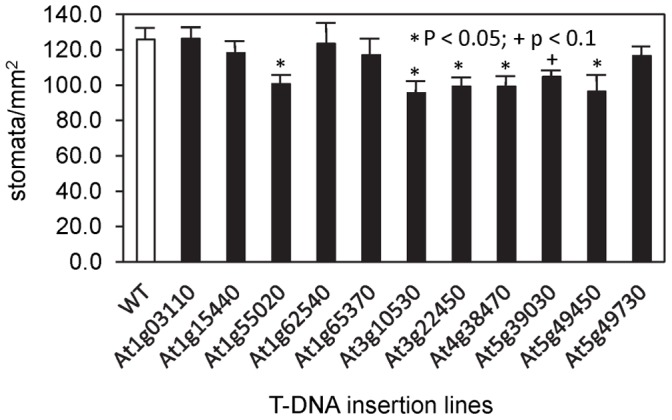
Stomatal density measurements on T-DNA insertion lines that showed impaired stomatal function. Measurements were made by direct observation of surface impressions of the abaxial side of mature leaves from vegetative rosettes. At least four leaves were examined for each mutant; the data are averaged values (± se). Samples marked with asterisks are significantly different from the wild type (Col-0) on the basis of a two-tailed *t*-test (p<0.05). The T-DNA insertion lines tested are as in Figure 5.

Sugar metabolism is distinctly different between guard cells and mesophyll cells. Mesophyll cells synthesize sugars by means of the photosynthetic carbon reduction cycle and export them to the phloem through the apoplast. Guard cells are deficient in the capacity of the carbon reduction cycle and must take up sugars from the apoplast; these sugars are metabolized glycolytically or are stored as starch (starch accumulates in guard cells in the day and is broken down at night). On the basis of their transcript levels, the major sugar transporters in the guard cell are the sucrose/H^+^ co-transporters *SUC1* and *SUC3* and the monosaccharide/H^+^ cotransporters *STP1* and *STP4*, but transcripts for *STP1* and *STP4* were 8- to 16-fold higher in guard cells than were those for *SUC1*, suggesting that sucrose arriving at the guard-cell wall is converted to monosaccharides by invertases and taken up as monosaccharides. *SUS3* was the most abundant sucrose synthase transcript in guard cells (*SUS3* transcripts were absent in the leaf as a whole); *SUS3* is probably responsible for the synthesis of ADP-glucose for starch synthesis in guard cells. The microarray data also showed that guard cells use different large subunits of ADP-glucose pyrophosphorylase (*APL4* and *APL3*) than do mesophyll cells (*APL1*), suggesting different regulation of starch synthesis in these two cell types. Trehalose is implicated in the regulation of carbohydrate metabolism [53], and transcripts for enzymes involved in trehalose metabolism are considerably more abundant in guard cells than the leaf. Four genes for trehalose phosphate synthase (TPS) enzymes were expressed at moderate to high levels in guard cells (*TPS1*, *TPS7*, *TPS8*, and *TPS11*); *TPS1* transcripts were 8-fold more abundant in guard cells than in the leaf, and *TPS8* and *TPS11* were up-regulated in guard cells but fall just below the 2-fold cutoff we used. Transcripts for trehalose phosphatases *TPPF* and *TPPG* and trehalase (*TRE*) were 8 to 30 fold more abundant in guard cells than in the leaf. These observations suggest an important role for trehalose metabolism in guard cells.

### Sugar-responsive Gene Expression in Guard Cells

Initially, we compared the effects of sucrose on guard cells from wild type Col-0 plants with those from the null mutant *rgs1–2*, because *RGS1 (REGULATOR OF G-PROTEIN SIGNALING 1)* is involved in sugar sensing in seedlings [22], [54]. Pair-wise comparisons of guard-cell transcripts revealed only three genes expressed differently in *rgs1* mutants and wild type Col plants: As expected, *RGS1* was low in the mutant regardless of sugar treatment; cytochrome c biogenesis protein (At1g49380) was down-regulated 3-fold by sucrose from the wild-type level, and a myb-family transcription factor (At2g40970) was 2-fold up-regulated by sucrose from the wild-type level.

In pair-wise comparisons of guard-cell gene expression profiles from sucrose-treated and mannitol-treated leaf strips of wild-type Col-0 plants, the steady-state level of 1769 transcripts were significantly different (*t*-test, p<0.05). Of these, 15 had a false discovery rate (FDR) <0.05, 51 had a FDR <0.1, and 136 had a FDR <0.2. The same comparison for intact guard cells from sucrose- and mannitol-treated leaf strips of the *rgs1* mutant revealed 858 genes with significant expression differences (*t*-test), and of these, 7 had a FDR <0.05, 44 had a FDR <0.1, and 165 had a FDR <0.2. Sucrose caused large effects on gene expression, but the variability among triplicate biological replicates was too large to reach a statistically-supported conclusion. Therefore, because the guard-cell transcript profiles for wild-type and *rgs1* plants were nearly identical, the data were reanalyzed after the wild-type and *rgs1* samples were pooled within each treatment. Considering the Col-0 and *rgs1* arrays as replicates, we found 2269 genes with at least a 2-fold response to sucrose (p<0.05), and 440 had a FDR of <0.05. In addition, 85% of the genes identified as sucrose responsive in the pairwise comparisons of triplicate biological replicates were present among the genes identified from the pooled data. Pooling the data therefore led to identification of largely the same set of sucrose-responsive genes but with much higher statistical significance.

Of the 440 sucrose-responsive genes, 244 were up-regulated by the sucrose treatment and 196 were down- regulated (Table 4 shows examples of sucrose-responsive guard-cell transcripts; the full list is given in Table S7). Figure 3 displays the functional categories of these sucrose-responsive genes. In broad overview, sucrose repressed expression of genes involved in photosynthesis, protein degradation, and sugar transport while up-regulating genes for starch, protein, nucleotide, and cell-wall synthesis, but expression of genes involved in a wide array of pathways including, transcription, signaling, hormone responses, redox maintenance, stress, and carbohydrate and lipid metabolism also changed.

Transcripts for seven genes involved in photosynthesis (including *PHOTSYSTEM 1 SUBUNIT N, PHOTOSYSTEM 1 SUBUNIT H1, CP12 DOMAIN-CONTAINING PROTEIN 2, PHOTOSYSTEM 2 5-kd protein,* and *PHOTOSYSTEM 2 SUBUNIT O*) were down-regulated 2- to 30-fold by sucrose. Many more genes involved in photosynthesis were down-regulated 1.5- to 1.9-fold by sucrose (data not shown). These observations are consistent with previous reports that sugars repress expression of photosynthetic genes [6], [55]. Expression of 63 genes encoding protein and nucleotide synthesis functions were up-regulated by sucrose (ribosomal proteins, initiation and elongation factors, nucleotide synthases), and expression of 12 genes involved in autophagy and ubiquitin-mediated protein degradation (starvation responses) were down-regulated. These observations are consistent with a general stimulatory effect of sugars on metabolism [16]–[18], [21].

Transcripts of genes involved in starch synthesis were up-regulated, including *APL3* (up 6.5-fold), the starch branching enzymes *SBE2.1* (up 4-fold) and *SBE2.2* (up 3-fold), an amylase *ISA3* (up 19-fold), and sucrose phosphate synthase 1F (*SPS1F*, up 2-fold). On the basis of our transcript data, *APL3* may be the major form of the large subunit of ADP glucose pyrophosphorylase found in guard cells in the presence of sucrose, and this enzyme carries out the rate-limiting step in starch biosynthesis [56]. The glucose-6-phosphate translocator *GPT2* was also strongly up-regulated (140-fold). This protein imports glucose-6-phosphate into plastids for starch synthesis in nongreen plant cells [57]. Previous studies have reported the up-regulation of *APL3* and *GPT2* expression by sugars [16], [58]. Sucrose also altered expression of a range of genes involved in carbohydrate metabolism and glycolysis, including two trehalose-phosphate synthases (*TPS5* was up-regulated 50-fold and *TPS11* down-regulated 10-fold), several invertases (*CINV2*, *FRUCT5 BFRUCT3* and a putative neutral invertase, At3g06500). *UGP1* (UDP glucose pyrophosphorylase, callose deposition) and pyruvate kinase were up-regulated, and *PFK1* (phosphofructokinase controls a key regulatory step in glycolysis) was down-regulated.

Sucrose altered expression of 23 genes for transporters; in particular seven genes for sugar transporters were affected, and all but two were down-regulated: *SUC1, STP4, GLT1*, and the putative sugar transporters At2g48020 and At4g36670 were down-regulated 2- to 4-fold, whereas *GPT2* and the sucrose efflux carrier *SWEET17* were up-regulated. Expression of the hexose transporter *STP1,* which is a highly abundant guard cell transcript, was unaffected. Other transporter genes affected by sucrose included the plasma-membrane aquaporin *TMP-C* (up 2-fold), mitochondrial transporters, and MATE-family and SEC14-family transporters, mostly up-regulated.

Genes encoding enzymes in many biosynthetic pathways showed mixed responses to sucrose (some up-regulated, some down-regulated, and many unaffected), but several showed consistent sucrose responses. Nine genes encoding proteins involved in cell-wall synthesis were up-regulated 2- to 4-fold by sucrose, including *CESA1* (CELLULOSE SYNTHASE 1). Genes involved in post-translational modifications (including the SnRK1 kinase subunit, *AKINβ1*, which Li et al. [59] have linked to sugar responses) and protein degradation (including *AUTOPHAGY8F*, *SERINE CARBOXYPEPTIDASE-LIKEL48,* and four different kelch repeat–containing F-box–family proteins) were down regulated by sucrose. Transcripts for several heat-shock proteins and chaperonins were up-regulated by sucrose (*HSP70*, *MTHSC70*, *ATJ13*, *HSP17.6*, *BIP2*, and one DNAJ domain–containing protein, At2g35720). Up-regulation of heat-shock proteins in response to sugars has been reported before [16], [21]. Our data also suggest that sucrose may alter the redox status of guard cells, as two thioredoxins (At5g61440, At1g11530), a glutaredoxin (At1g03850), and a sulfiredoxin (SRX) were down-regulated. Redox changes have been implicated in the regulation of starch biosynthesis [60].

Twenty-seven genes involved in transcription and RNA processing were modulated by sucrose, as well as 14 genes involved in signaling; these genes could affect stomatal movements. These include 15 transcription factors, two histone deacetylases, a histone acetylase, a histone methyltransferase, four G proteins, and proteins involved in calcium, GA, jasmonate, and phosphoinositide signaling. The most strongly sucrose responsive were the transcription factor *BZIP1* (down-regulated 30-fold; Kang et al. [61] have linked this gene to sugar signaling), *HD2A* (*HISTONE DEACETYLASE 2A*, up 25-fold), *NOP56* (HOMOLOG OF NUCLEOLAR PROTEIN NOP56, up 10-fold) *PAP2* (*PRODUCTION OF ANTHOCYANIN PIGMENT 2*, up 12-fold), the G-proteins *RACK1B* and *RACK1C* (both up 8-fold), and the map kinase kinase *MKK7* (up 10-fold). In addition, two genes involved in lipid metabolism, and potentially signaling, were affected: the phosphoethanolamine methyltransferase *NMT3* (up 20-fold) and a lipoxygenase (*LOX1*, down 13-fold).

### Confirmation of the Microarray Data by RT-PCR

Twelve sugar-responsive genes were chosen for real-time PCR (RT-PCR) to confirm the microarray data; the results are shown in Figure 4. The difference in transcript levels between RNAs of guard cells from sucrose-treated and mannitol-treated leaf strips was in the same direction (up- or down-regulated by sucrose) for all 12 genes, and except for *SUC1*, the differences were statistically significant (*t*-test, p<0.05). Of all the genes tested, *SUC1* differed the least (3-fold), and the *t*-test on the RT-PCR data just missed significance (p = 0.06). The magnitude of the changes measured by the two techniques agreed within a factor of 2.5 for 7 of the 12 genes tested, but some genes showed larger differences.

### Testing Candidate Genes for Defects in Stomatal Closure

From among the sucrose-responsive genes in guard cells, 50 were chosen that showed at least a 4-fold change in expression and for which T-DNA insertion lines were available through public stocks. Plants of these lines were grown to maturity (∼50 days on 10 h light/14 h dark cycles), and stomatal closure was induced by reduction of the RH (from 60–70% to less than 20%) and by increase of [CO_2_] (from 390 ppm to 800 ppm). Conductance and photosynthetic rates were measured before induction of stomatal closure, the rate of closure (slope of conductance change) was measured, and conductance was measured after closure. Twelve lines were identified in which defects were detected (listed in Table 5). Plants from these lines were self-pollinated, and the next generation of plants was tested in the same ways (Figures 5–7). Nine lines had lower steady-state conductance than did the wild type (Figure 5A), and five of these also had lower steady-state photosynthetic rates (Figure 5B). Three insertion lines (*tps5-1* and two protein kinases At4g38470, At5g39030) showed lower water-use efficiency than wild type at steady state under high RH and after the increase in [CO_2_] (Figure 6A, B), and all of the insertion lines except *tps5-1* had higher water-use efficiency than did the wild type at the lower RH and with higher [CO_2_] (Figure 6C). Six lines also differed significantly from the wild type in the rate of conductance change upon RH decrease (Figure 7B). In summary, all 12 of the tested insertion lines showed significant effects in at least two of the tests of stomatal function, and 7 of the lines were significantly different than wild type in a majority of the tests.

All of the lines appeared phenotypically normal, but because stomatal density affects conductance, stomatal density was measured for all 12 lines (Figure 8). Five had stomatal densities 20–25% lower than that of the wild type (those marked by asterisks in Figure 8). That of a sixth line, insertion in At5g39030, was also lower than that of the wild type but did not reach the significance threshold (p<0.05). The stomatal density of *tps5-1* plants was determined in a separate experiment and was found to be the same as that of the wild type (data not shown). Aside from the reduction of stomatal density in some lines, no defects were observed in the morphology of the guard cells or epidermal cells.

Although we tested only a single allele for each gene, and the lines were not determined to be null alleles (although the insertion in *TPS5*, resulting in *tps5-1*, is a known null allele [62]), all 12 of the identified genes could have roles in stomatal function. However, *TPS5* (At4g17770), the TRAF domain-containing protein (At1g65370) and the WD repeat–containing protein (At1g15440) are of particular interest. The insertion lines for these genes showed effects in nearly every test of stomatal function without a change in stomatal density, and these genes have potential roles in cell signaling. *TPS5* plays a role in thermal tolerance in *Arabidopsis* [62], and trehalose metabolism may have a regulatory role in carbohydrate metabolism [53], which could link sugars and sugar sensing to stomatal movements. The annotation for At1g15440 (PERIODIC TRYPTOPHAN PROTEIN 2) indicates it contains a CUL4-RING domain, which is found in ubiquitin ligases. So, this gene could be involved in response regulation through protein degradation. At1g65370 contains a TRAF domain involved in cytokine signaling through self-oligomerization and binding to cytokine receptors [63]. In *Arabidopsis,* some TRAF domain proteins interact with AP domain containing transcription factors [64].

### Conclusions

Combining the published microarray data on guard-cell protoplasts with those obtained from guard cells isolated by dissection provides a robust view of the *Arabidopsis* guard-cell transcriptome. Nearly all the genes previously identified as important in stomatal functioning are present in the transcriptome of intact guard cells providing a high level of confidence. However, additional genes were uncovered here using intact guard cells compared to gene profiling that used guard cell protoplasts. The guard-cell transcriptome is particularly rich in transcription factors, so modulation of transcription may be important in guard-cell responses. Transcripts for signaling proteins, and protein modification/degradation, are also abundant, suggesting the possible existence of unknown effectors of guard-cell movements and associated signaling pathways. The functional role of these genes in guard-cell biology provides fertile ground for further investigation. Toward that end, we extended previous studies of guard-cell gene profiles by showing that in intact guard cells, sugar regulates an informative set of genes. We identified both transcription factors and signaling-protein genes that are sucrose responsive and that could connect sucrose signaling to guard-cell movements. Although the data presented here do not prove unequivocally that sucrose has a signaling role in guard cells, the effects of the knockouts of sucrose-responsive genes lends support to this hypothesis and provides an avenue for future studies.

## Supporting Information

Figure S1 A. Density plot. Histograms of the raw log2 signal intensities across each of the 18 microarrays of amplified guard cell RNAs. All arrays had similar intensities, none were bi- or trimodal. B. RNA degradation plot. Affymetrix RNA degradation plots for the amplified guard cell RNAs. All 18 microarrays show similar slopes from 5′ to 3′ on the control genes on the array, indicating consistent RNA quality. The second round of RNA amplification shortens the RNAs, increasing the slope on these plots compared with what is typically observed.
(TIFF)

Figure S2 **Scatter plots comparing normalized signal intensities for each data point on biological replicate microarrays.** The lines show the range for two-fold signal intensity differences. A,C,E are arrays for RNAs from guard cells of wild type (Col-0) plants. B,D,E are arrays of guard cell RNAs from *rgs1* plants. A,B are arrays of RNAs from leaves treated with mannitol. C,D are arrays of RNAs from leaves treated with sucrose. E,F are arrays of RNAs from leaves floated on buffer without added sugar (NS).
(PDF)

Figure S3 **Graphical display from MapMan of the categories of genes that are differentially expressed in guard cells versus the leaf, the data are those in Table S4.** Blue symbols indicate genes up-regulated in guard cells and red symbols indicate genes that are up-regulated in the leaf. Categories that were significantly different (Wilcoxon rank sum test, Benjamini Hochberg corrected) are #1 (photosynthesis), #27 (transcription factors and RNA modifying enzymes), #34 (transporters), #9 (mitocondrial electron transport), #21 (redox), and #19 (tetrapyrrole synthesis). #30 was near significance (p = 0.1). #29 is protein synthesis and in this category most of the red symbols are genes for ribosomal proteins (chloroplast and cytoplasmic), most of the blue symbols are genes for protein modifying enzymes.
(TIFF)

Table S1 **Primers used for RT-PCR.**
(DOCX)

Table S2 **Reproducibility and linearity of T7 RNA polymerase RNA amplification.** RNA was isolated from a sample of fresh *Arabidopsis* leaves and divided into two subsamples. A 500-ng aliquot of each subsample was used for cDNA synthesis followed by RT-PCR that determined the levels of transcripts of the genes listed (Unamplified). Then a 500-ng aliquot of each RNA subsample was amplified twice with T7 RNA polymerase, and the levels of transcripts were assayed by RT-PCR after each round of RNA amplification. The results are expressed as the average obtained for the two subsamples +/− the range.
(DOC)

Table S3 **Transcripts that differed by 2-fold or more between intact guard cells and guard cell protoplasts (t-test p<0.05, FDR<0.05).** Signal intensities are normalized log2 values.
(XLS)

Table S4 **Genes that were differentially expressed (2-fold or more) in guard cells versus leaves.** All differences are statistically significant (t-test p<0.05, FDR<0.05, calls present in at least one group (either guard cells or leaves)). Positive fold differences indicate preferential expression in guard cells. Signal intensities are normalized log2 values.
(XLS)

Table S5 **List of all transcripts detected in guard cells.** Affymetrix quality calls for each array were converted to numerical values (calls present = 1, calls marginal = 0.5, calls absent = 0). The signal intensities are normalized log2 values. The call values shown are averages across all the arrays in each group (leaves or guard cells).
(XLS)

Table S6 **The ABC transporters that are more highly expressed in guard cells than in the leaf.**
(XLS)

Table S7 **Genes in guard cells that responded to sucrose (2-fold or greater difference, FDR <0.05).** Positive fold changes indicate the gene is up-regulated in sucrose compared with the mannitol control, negative fold changes indicate down-regulation in sucrose compared with mannitol. Fold expression in mannitol is the relative level of expression of each gene compared with the average level of expression of all genes (present calls only) in all the arrays of samples treated with mannitol. This information is provided to show whether the transcripts for a given gene are abundant or rare.
(XLS)
