# Supplementary figures and images for: A Comparative Study of the Arabidopsis thaliana Guard-Cell Transcriptome and Its Modulation by Sucrose
Source: PLoS One. 2012 Nov 21;7(11):e49641. doi: 10.1371/journal.pone.0049641 (PMC3504121; doi:10.1371/journal.pone.0049641)

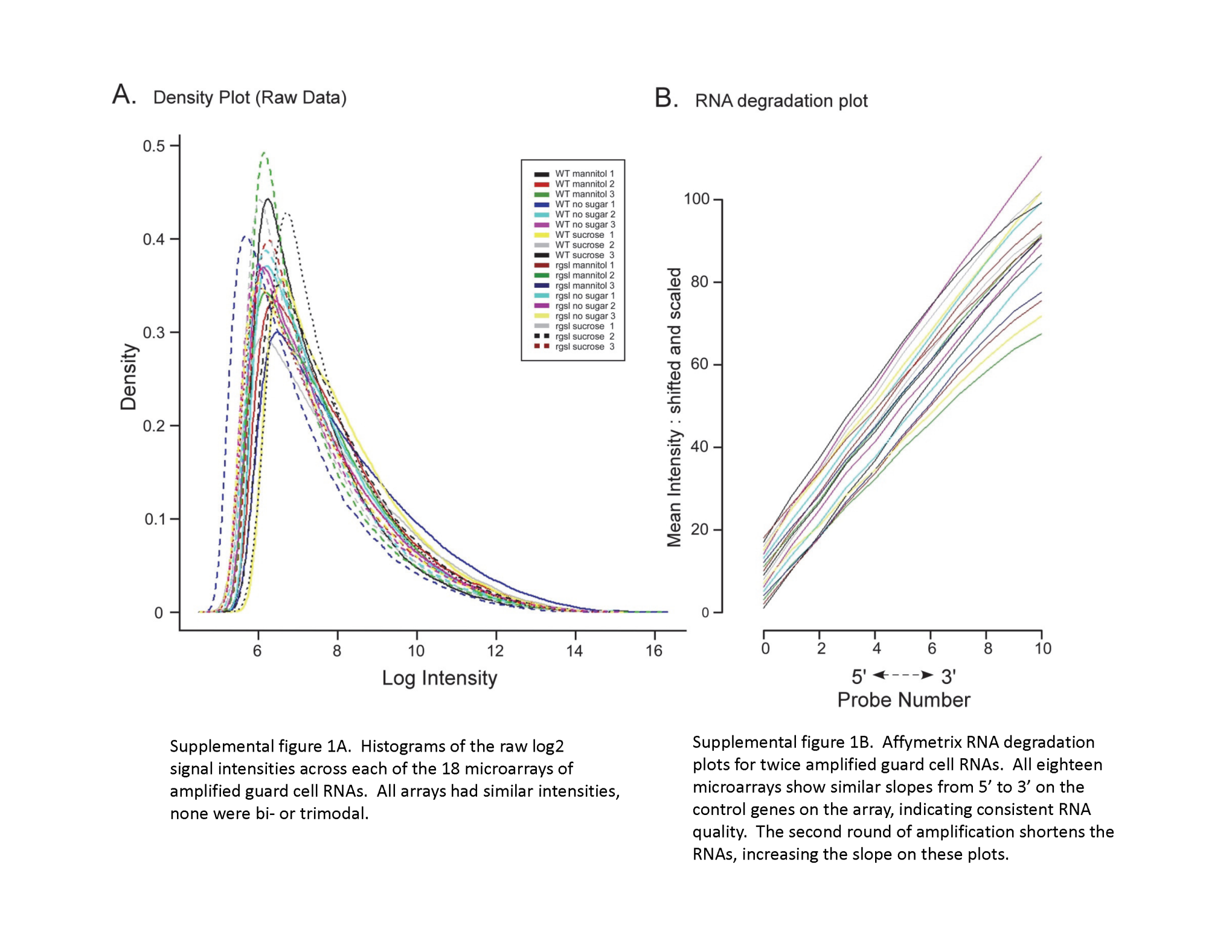

Supplement: Figure S1 — A. Density plot. Histograms of the raw log2 signal intensities across each of the 18 microarrays of amplified guard cell RNAs. All arrays had similar intensities, none were bi- or trimodal. B. RNA degradation plot. Affymetrix RNA degradation plots for the amplified guard cell RNAs. All 18 microarrays show similar slopes from 5′ to 3′ on the control genes on the array, indicating consistent RNA quality. The second round of RNA amplification shortens the RNAs, increasing the slope on these plots compared with what is typically observed. (TIFF) [file pone.0049641.s001.tif]

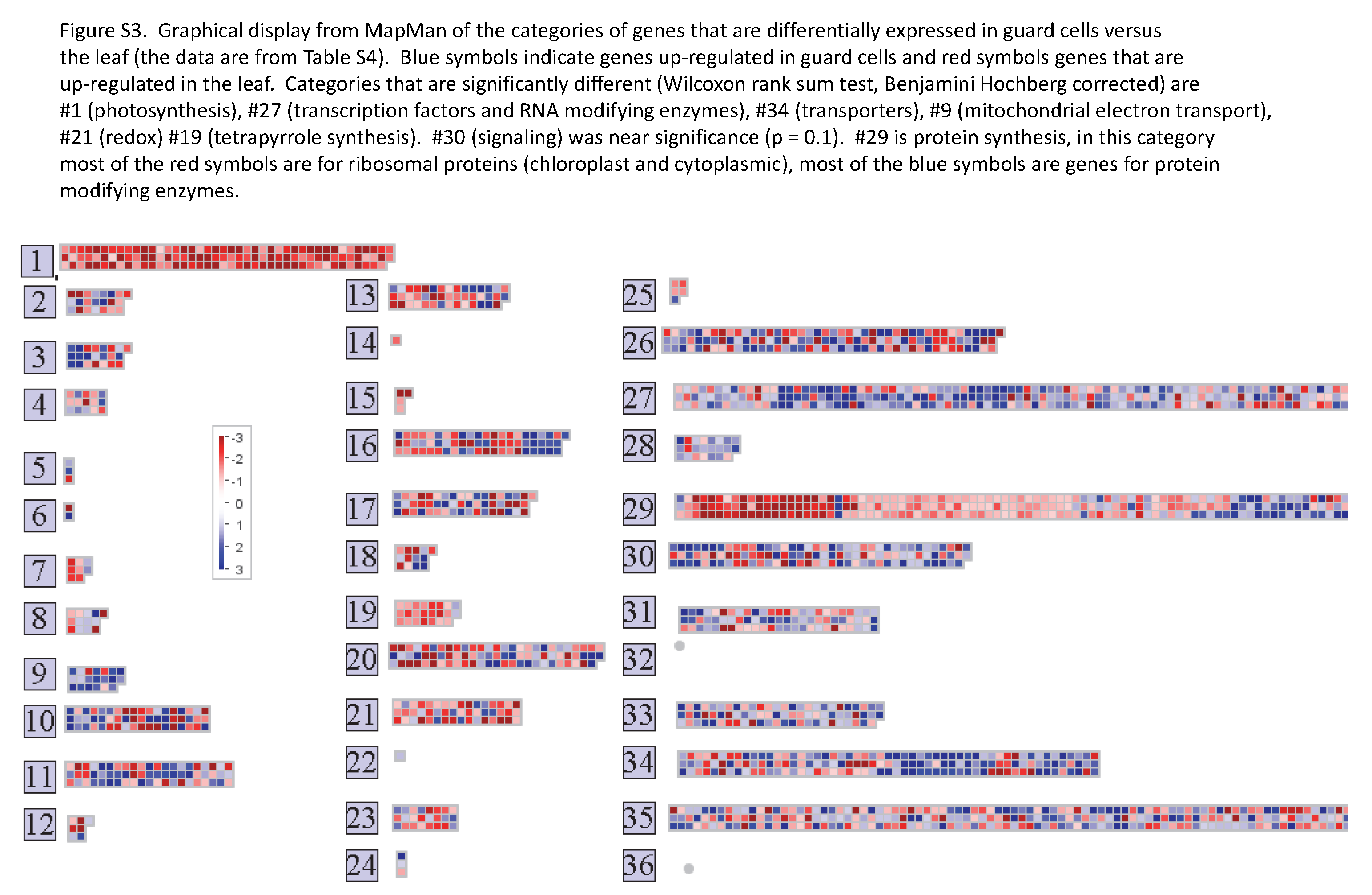

Supplement: Figure S3 — Graphical display from MapMan of the categories of genes that are differentially expressed in guard cells versus the leaf, the data are those in Table S4. Blue symbols indicate genes up-regulated in guard cells and red symbols indicate genes that are up-regulated in the leaf. Categories that were significantly different (Wilcoxon rank sum test, Benjamini Hochberg corrected) are #1 (photosynthesis), #27 (transcription factors and RNA modifying enzymes), #34 (transporters), #9 (mitocondrial electron transport), #21 (redox), and #19 (tetrapyrrole synthesis). #30 was near significance (p = 0.1). #29 is protein synthesis and in this category most of the red symbols are genes for ribosomal proteins (chloroplast and cytoplasmic), most of the blue symbols are genes for protein modifying enzymes. (TIFF) [file pone.0049641.s003.tif]
