# Supplementary material for: A Comparative Study of the Arabidopsis thaliana Guard-Cell Transcriptome and Its Modulation by Sucrose
Source: PLoS One. 2012 Nov 21;7(11):e49641. doi: 10.1371/journal.pone.0049641 (PMC3504121; doi:10.1371/journal.pone.0049641)
Supplement: Figure S2 — Scatter plots comparing normalized signal intensities for each data point on biological replicate microarrays. The lines show the range for two-fold signal intensity differences. A,C,E are arrays for RNAs from guard cells of wild type (Col-0) plants. B,D,E are arrays of guard cell RNAs from rgs1 plants. A,B are arrays of RNAs from leaves treated with mannitol. C,D are arrays of RNAs from leaves treated with sucrose. E,F are arrays of RNAs from leaves floated on buffer without added sugar (NS). (PDF) [file pone.0049641.s002.pdf]

Figure S2. Scatter plots comparing normalized signal intensities for each data point on biological replicate microarrays. The lines show the range for two-fold signal intensity differences. A, C, E Arrays for RNAs from guard cells of wild type (Col-0) plants. B, D, E Guard cell RNAs from rgs1 plants. A, B leaves treated with mannitol. C, D leaves treated sucrose. E, F leaves floated on buffer without added sugar (NS).

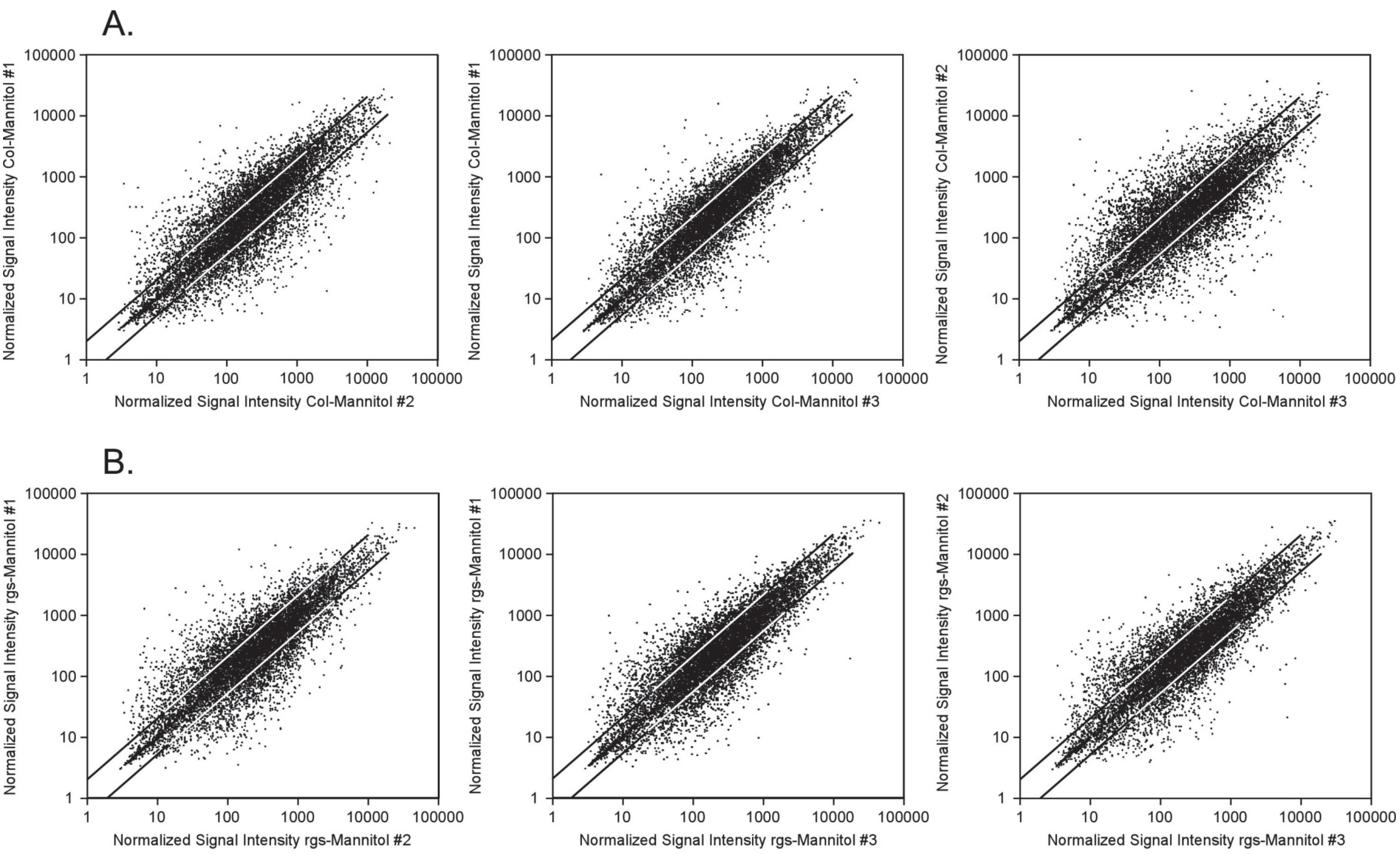

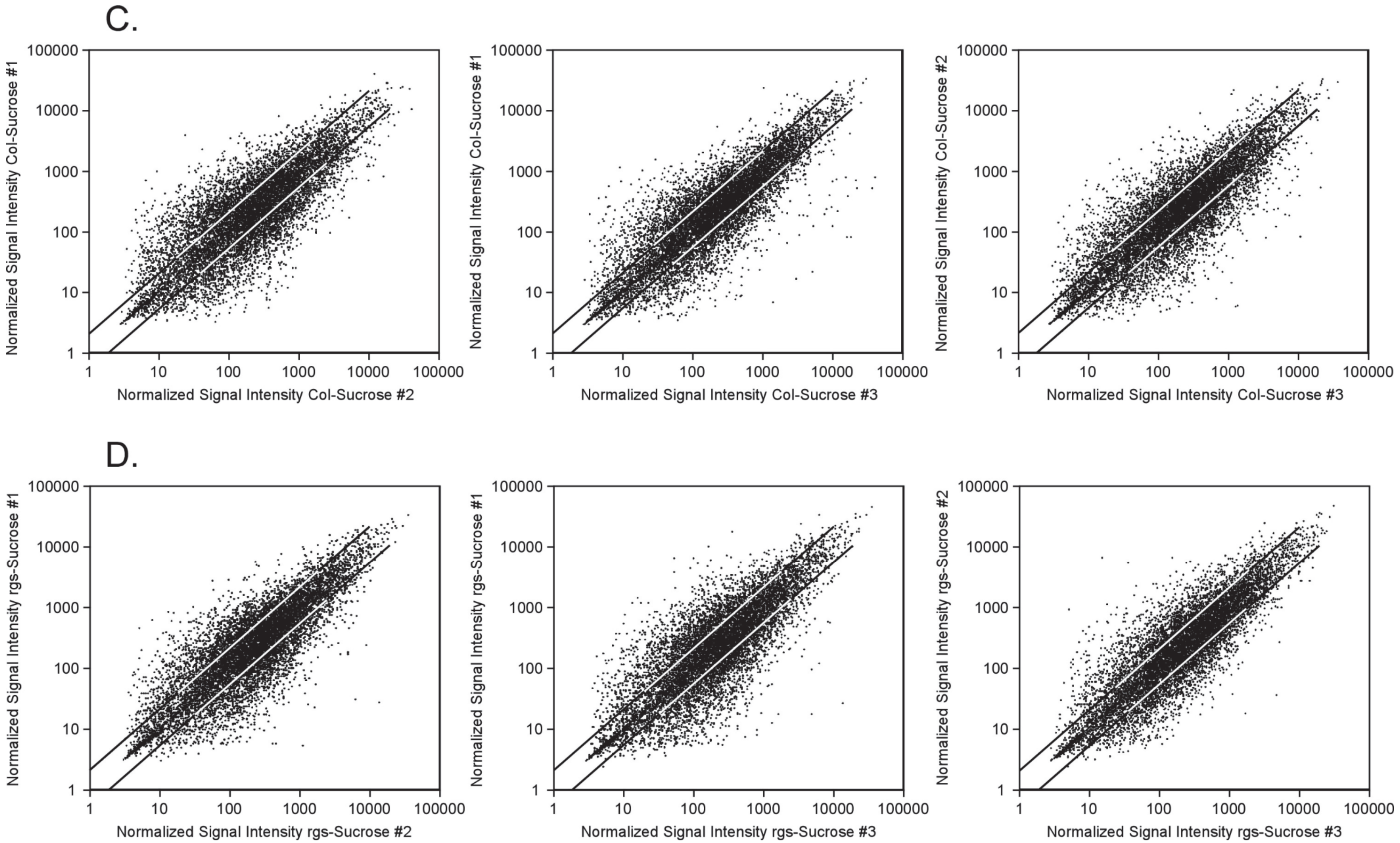
C.
D.
Normalized Signal Intensity Col-Sucrose #1
Normalized Signal Intensity Col-Sucrose #2
Normalized Signal Intensity Col-Sucrose #1
Normalized Signal Intensity Col-Sucrose #3
Normalized Signal Intensity Col-Sucrose #2
Normalized Signal Intensity Col-Sucrose #3
Normalized Signal Intensity rgs-Sucrose #1
Normalized Signal Intensity rgs-Sucrose #2
Normalized Signal Intensity rgs-Sucrose #1
Normalized Signal Intensity rgs-Sucrose #3
Normalized Signal Intensity rgs-Sucrose #2
Normalized Signal Intensity rgs-Sucrose #3
1
10
100
1000
10000
100000

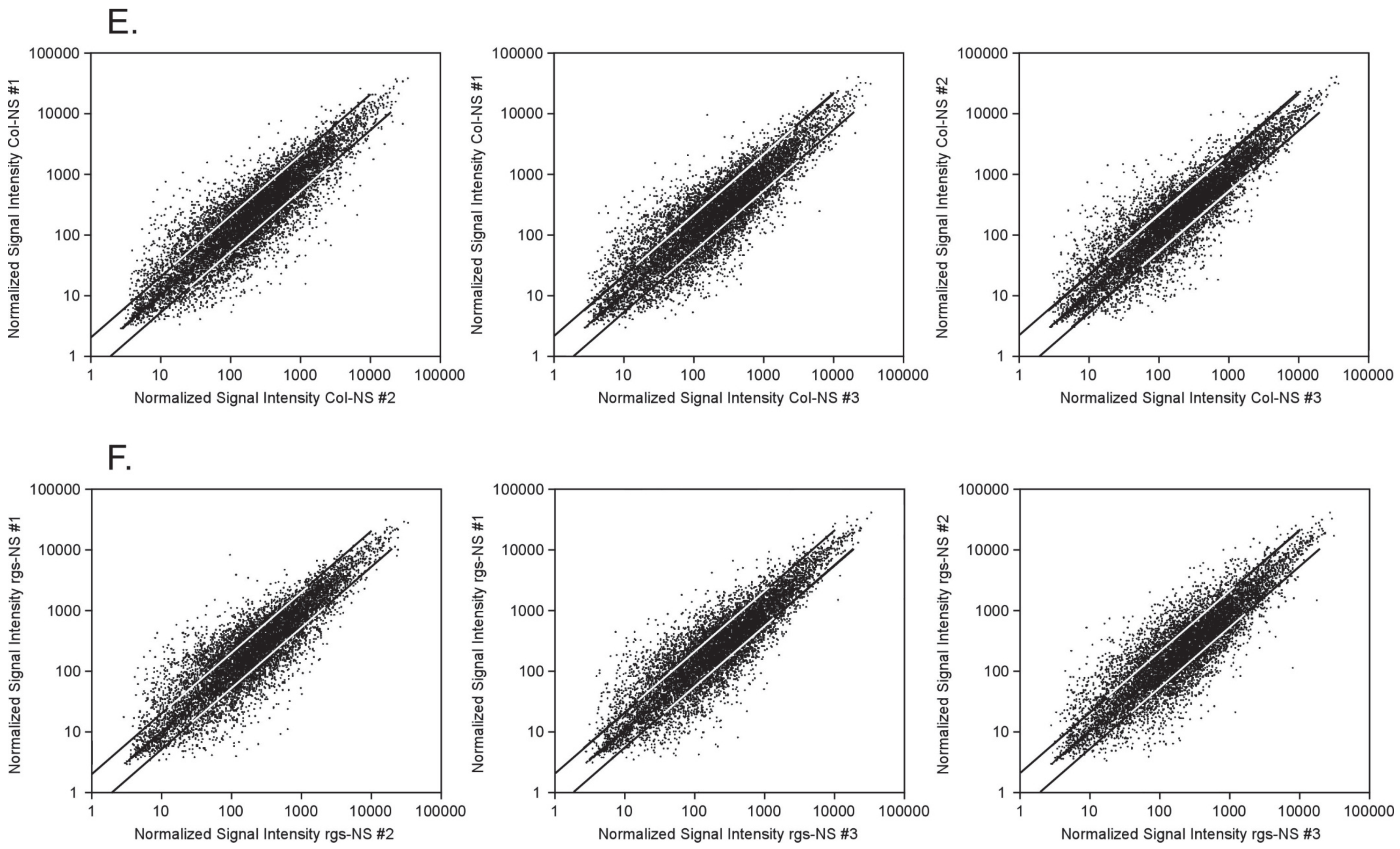
E.
Normalized Signal Intensity Col-NS #1
Normalized Signal Intensity Col-NS #2
Normalized Signal Intensity Col-NS #1
Normalized Signal Intensity Col-NS #3
Normalized Signal Intensity Col-NS #2
Normalized Signal Intensity Col-NS #3
F.
Normalized Signal Intensity rgs-NS #1
Normalized Signal Intensity rgs-NS #2
Normalized Signal Intensity rgs-NS #1
Normalized Signal Intensity rgs-NS #3
Normalized Signal Intensity rgs-NS #2
Normalized Signal Intensity rgs-NS #3
1
10
100
1000
10000
100000
