# Supplementary material for: A Comparative Study of the Arabidopsis thaliana Guard-Cell Transcriptome and Its Modulation by Sucrose
Source: PLoS One. 2012 Nov 21;7(11):e49641. doi: 10.1371/journal.pone.0049641 (PMC3504121; doi:10.1371/journal.pone.0049641)
Supplement: Table S1 — Primers used for RT-PCR. (DOCX) [file pone.0049641.s004.docx]

Table S1

Primers used for RT-PCR

HD2A At3g44750

Forward 5’CAACTCAGGGAATGCACTTG 3’

Reverse 5’GGCAAAATTCCATAGAAATCACA 3’

ISA3 At4g09020

Forward 5’TTTTGTGAGGGAAGGTGTGG 3’

Reverse 5’CCTCTACCGTGCGTTATCGT 3’

APL3 At4g39210

Forward 5’ACTGTGGTTGTCGAAAAGGC 3’

Reverse 5’TTTAACTTCCGGCCAAACAC 3’

BXL1 At5g49360

Forward 5’GGA TCA CAT GCA AAC AGT GG 3’

Reverse 5’GCA CTA TTG ATC GGC AAC AA 3’

SEN1 At4g35770

Forward 5’GAA TGA GCT GCC GGT AGA AG 3’

Reverse 5’TGA TGA TTG ATA CTT GCG TTG A 3’

CCH1 At5g13630

Forward 5’CAG AGA GAT GAT GTC GTT TGG 3’

Reverse 5’ TGG AAG ACA AGA TCG AAG GG 3’

BGAL4 At5g56870

Forward 5’TCG ACG CAA AGA AAT GCT TA 3’

Reverse 5’TTC CGT TAG GAT CAC CAC CT 3’

bZIP1 At5g49450

Forward 5’TGG CTC TCG AGT TAC GTT AGC 3’

Reverse 5’ AAT CTC CAA CCG CTA TTC CC 3’

LOX1 At1g55020

Forward 5’GAG AGG AAC GAC GAC GAG AC 3’

Reverse 5’ACG CTA TTT GGA ATT CCC CT 3’

GPT2 At1g61800

Forward 5’CGA AGC AGT GAG GAT GGT TT 3’

Reverse 5’TCA CCG GAA TGT TCT CTC CT 3’

SUC1 At1g71880

Forward 5’CATCCATATTCTCAAGCTGCTC 3’

Reverse 5’GCTAATACTCCACTAATCGCC 3’

TPS5 At4g17770

Forward 5’TCTGATGCTCCTTCTTCCGT 3’

Reverse 5’AGCTGCAAGAGAAGCGAGTC 3’

TPS11 At2g18700

Forward 5’ TTGGAGAGCGTACACGACTG 3’

Reverse5’ AAGTGGGCAAAATCATGAGG 3’

ACT2 At3g18780

Forward 5’ ATCCCTCAGCACCTTCCAAC 3’

Reverse 5’ ACAAACTCACCACCACGAAC 3’

APL4 At2g21590

Forward 5’ ATTGGTGAACGATCACGTC 3’

Reverse 5’-ATTGGAACCTTTCCTTCTGC 3’

RBCS At1g67090

Forward 5’ ACTCACCCGGATACTATGATG 3’

Reverse 5’ CACTCTTCCACTTCCTTCAAC 3’

KAT1 At5g46240

Forward 5’ AGCAACCAAATCATCAAGCC 3’

Reverse 5’ CAGCCTCCAAACTTCTCAC 3’

HAB1 At1g72770

Forward 5’ GAAATAGCAAGGAGACGG 3’

Reverse 5’ GTAGAGCAAGCATTGAGAGG 3’
