# Supplementary material for: A Comparative Study of the Arabidopsis thaliana Guard-Cell Transcriptome and Its Modulation by Sucrose
Source: PLoS One. 2012 Nov 21;7(11):e49641. doi: 10.1371/journal.pone.0049641 (PMC3504121; doi:10.1371/journal.pone.0049641)
Supplement: Table S2 — Reproducibility and linearity of T7 RNA polymerase RNA amplification. RNA was isolated from a sample of fresh Arabidopsis leaves and divided into two subsamples. A 500-ng aliquot of each subsample was used for cDNA synthesis followed by RT-PCR that determined the levels of transcripts of the genes listed (Unamplified). Then a 500-ng aliquot of each RNA subsample was amplified twice with T7 RNA polymerase, and the levels of transcripts were assayed by RT-PCR after each round of RNA amplification. The results are expressed as the average obtained for the two subsamples +/− the range. (DOC) [file pone.0049641.s005.doc]

Table S2. Reproducibility and linearity of T7 RNA polymerase RNA amplification. RNA was isolated from a sample of fresh *Arabidopsis* leaves and divided into two subsamples. A 500-ng aliquot of each subsample was used for cDNA synthesis followed by RT-PCR that determined the levels of transcripts of the genes listed below (Unamplified). Then a 500-ng aliquot of each RNA subsample was amplified twice with T7 RNA polymerase, and the levels of transcripts were assayed by RT-PCR after each round of RNA amplification. The results are expressed as the average obtained for the two subsamples +/- the range.

| RNA Treatment | Gene Assayed | ng Transcript/ng RNA  In Original Sample | Fold Amplification Over Previous Step | Overall Amplification |
| --- | --- | --- | --- | --- |
| Unamplified | ACT2 | 2.2 x 10^-7^ +/- 1.9 x 10^-8^ |  |  |
| Amplified Once |  | 3.4 x 10^-4^ +/- 7.6 x 10^-5^ | 1,515 +/-225 |  |
| Amplified Twice |  | 2.8 x 10^-1^ +/- 6.0 x 10^-3^ | 872 +/- 178 | 1.3 x 10^6^ |
|  |  |  |  |  |
| Unamplified | HAB1 | 1.1 x 10^-7^ +/- 6.0 x 10^-9^ |  |  |
| Amplified Once |  | 1.4 x 10^-4^ +/- 1.5 x 10^-5^ | 1,280 +/- 70 |  |
| Amplified Twice |  | 1.1 x 10^-1^ +/- 1.9 x 10^-2^ | 801 +/- 54 | 1.0 x 10^6^ |
|  |  |  |  |  |
| Unamplified | RBCS | 3.1 x 10^-5^ +/- 4.4 x 10^-6^ |  |  |
| Amplified Once |  | 7.8 x 10^-2^ +/- 6.1 x 10^-3^ | 2,565 +/-165 |  |
| Amplified Twice |  | 2.4 x 10^1^ +/- 1.3 | 312 +/- 41 | 0.8 x 10^6^ |
|  |  |  |  |  |
| Unamplified | KAT1 | 1.3 x 10^-8^ +/- 1.2 x 10^-9^ |  |  |
| Amplified Once |  | 1.5 x 10^-5^ +/- 3.0 x 10^-7^ | 1170 +/- 80 |  |
| Amplified Twice |  | 1.3 x 10^-2^ +/- 3.7 x 10^-3^ | 834 +/- 226 | 1.0 x 10^6^ |
|  |  |  |  |  |
| Unamplified | APL4 | 7.3 x 10^-8^ +/- 8.0 x 10^-10^ |  |  |
| Amplified Once |  | 7.5 x 10^-5^ +/- 1.9 x 10^-5^ | 1022 +/- 248 |  |
| Amplified Twice |  | 2.8 x 10^-2^ +/- 1.1 x 10^-2^ | 359 +/- 52 | 0.4 x 10^6^ |
|  |  |  |  |  |
| Unamplified | SUC1 | 4.2 x 10^-8^ +/- 3.1 x 10^-9^ |  |  |
| Amplified Once |  | 4.3 x 10^-5^ +/- 2.8 x 10^-6^ | 1038 +/- 142 |  |
| Amplified Twice |  | 2.6 x 10^-2^+/- 1.6 x 10^-3^ | 589 +/- 1 | 0.6 x 10^6^ |
